# Supplementary material for: Psychosocial impact of COVID-19 pandemic on front-line healthcare workers in Sierra Leone: an explorative qualitative study
Source: BMJ Open. 2023 Aug 22;13(8):e068551. doi: 10.1136/bmjopen-2022-068551 (PMC10445370; doi:10.1136/bmjopen-2022-068551)
Supplement: Supplementary data [file bmjopen-2022-068551supp001.pdf]

Topic Guide

A. Preliminary Note

This is the initial idea for the interview topic guide, and this will be extended and adjusted with growing insight into the research topic throughout the course of the research due to the iterative nature of qualitative research.

B. Introductions

Thank you very much for taking the time for this interview today. As outlined in the information sheet, our study aims to explore the psychosocial impacts of the COVID-19 pandemic and the Ebola outbreak on healthcare workers in Freetown Sierra Leone and develop recommendations for appropriate support. I am especially interested in your views and experiences as a frontline healthcare worker. Do you have any questions before we begin the interview?

C. Questions

1. Life History

- Would you tell be a bit about your professional background?
- What is your cadre as a healthcare worker?
- Are you employed by the government or by a non-governmental organization?
- What position do you hold in the organization?
- How long have you been working in the current COVID-19 response?
- What department did you working in prior to the COVID-19 response?

2. Work Environment

- Please tell me about your experiences in taking care of patients with COVID-19?
- How did you feel (emotions) on the first day?
- How are you feeling today?
- How do you feel when you get to work?
- What facilitates/ motivates you to give care to these patients?
- How prepared to you feel to provide care for COVID-19 patients?
- What challenges (emotional and resources) have you faced while providing care in the unit?
- How have you responded to the emotional challenges? (coping mechanisms)
- How has interactions with fellow healthcare workers been affected by working in the unit?
- What support (mental health/ psychological support) have you received?
- How do you feel about the support you have received? (assessible, efficient, preferences, shared decision making)
- How could the service provided be improved?

- Are there any other support (mental health /psychological support) would you have liked to have access to?
- Do you have any management roles/responsibilities? **(If No, skip to section 3)**
- Please tell me about your management experiences during COVID-19?
- How has COVID-19 pandemic affected your managerial duties?
- How has COVID-19 pandemic affected healthcare service provision within the facility?
- How prepared do you feel healthcare workers are to provide care for COVID-19 patients?
- What measures have you put in place to ensure healthcare workers are prepared to provide care for COVID-19 patients?
- What challenges (emotional and resources) do you feel frontline healthcare workers are facing while providing care in the unit?
- What challenges (emotional and resources) have frontline healthcare expressed?
- What do you feel is responsible for the different emotions' healthcare workers have expressed?
- How have you responded to the emotional challenges?
- What support (mental health/ psychological support) have you provided?
- How do you feel these support services have been received? (accessible/efficient/preference /shared decision making)
- How could the service provided be improved?
- Are there any other support (mental health /psychological support) would you like healthcare workers to have access to?
- Do you feel healthcare workers involved in the COVID-19 response should be recognized and appreciated? How?

3. *Family and Community?*

- Has working in this unit affected your interactions with family/ friends/ community?
- How has your family and community reacted to your work with COVID-19 patients?
- How have you responded to their reactions?
- Do you receive any support have from your family/ friends/community?

4. *Prior experience in outbreak?*

- Have you had any prior experience providing care/ in a managerial position during the Ebola outbreak?
- Can you share how you felt (experiences) while taking care of patients with Ebola
- How would you compare your emotional response providing care during Ebola to COVID-19 (similarities/differences)?
- Do you feel prior experience/exposure gained during the Ebola outbreak influence your reactions and feeling during the COVID-19 pandemic?

D. Concluding the interview

- Is there anything you would like to add to this discussion, or anything that we may have missed?
- *Give a recap of the discussion.*
- Do you have any questions you would like to ask me?
- Thank you very much for this interview and your time.
