# Supplementary material for: Psychosocial impact of COVID-19 pandemic on front-line healthcare workers in Sierra Leone: an explorative qualitative study
Source: BMJ Open. 2023 Aug 22;13(8):e068551. doi: 10.1136/bmjopen-2022-068551 (PMC10445370; doi:10.1136/bmjopen-2022-068551)
Supplement: Author's manuscript [file bmjopen-2022-068551.draft_revisions.pdf]

BMJ Open is committed to open peer review. As part of this commitment we make the peer review history of every article we publish publicly available.

When an article is published we post the peer reviewers' comments and the authors' responses online. We also post the versions of the paper that were used during peer review. These are the versions that the peer review comments apply to.

The versions of the paper that follow are the versions that were submitted during the peer review process. They are not the versions of record or the final published versions. They should not be cited or distributed as the published version of this manuscript.

BMJ Open is an open access journal and the full, final, typeset and author-corrected version of record of the manuscript is available on our site with no access controls, subscription charges or pay-per-view fees (http://bmjopen.bmj.com).

If you have any questions on BMJ Open's open peer review process please email info.bmjopen@bmj.com

BMJ Open

The Psychosocial Impact of COVID-19 Pandemic on Frontline Healthcare Workers in Sierra Leone: We Need To Protect Our Healthcare Workers!

Journal:	*BMJ Open*
Manuscript ID	bmjopen-2022-068551
Article Type:	Original research
Date Submitted by the Author:	24-Sep-2022

Complete List of Authors:	Tengbe, Sia Morenike; Sierra Leone Ministry of Health and Sanitation<br>Kamara, Ibrahim Franklyn; World Health Organisation Country Office for Sierra Leone, Health Security and Emergency Cluster<br>Ali, Desta B; Sierra Leone Ministry of Health and Sanitation<br>Koroma, Fanny F; Sierra Leone Ministry of Health and Sanitation<br>Sevalie, Stephen; 34 Military Hospital, Wilberforce; Case Management Pillar, National COVID-19 Emergency Response Centre,<br>Dean, Laura; Liverpool School of Tropical Medicine, International Public Health<br>Theobald, Sally ; Liverpool School of Tropical Medicine, International Public Health

Keywords:	COVID-19, Public health < INFECTIOUS DISEASES, MENTAL HEALTH

SCHOLARONE™
Manuscripts

I, the Submitting Author has the right to grant and does grant on behalf of all authors of the Work (as defined in the below author licence), an exclusive licence and/or a non-exclusive licence for contributions from authors who are: i) UK Crown employees; ii) where BMJ has agreed a CC-BY licence shall apply, and/or iii) in accordance with the terms applicable for US Federal Government officers or employees acting as part of their official duties; on a worldwide, perpetual, irrevocable, royalty-free basis to BMJ Publishing Group Ltd ("BMJ") its licensees and where the relevant Journal is co-owned by BMJ to the co-owners of the Journal, to publish the Work in this journal and any other BMJ products and to exploit all rights, as set out in our licence.

The Submitting Author accepts and understands that any supply made under these terms is made by BMJ to the Submitting Author unless you are acting as an employee on behalf of your employer or a postgraduate student of an affiliated institution which is paying any applicable article publishing charge ("APC") for Open Access articles. Where the Submitting Author wishes to make the Work available on an Open Access basis (and intends to pay the relevant APC), the terms of reuse of such Open Access shall be governed by a Creative Commons licence – details of these licences and which Creative Commons licence will apply to this Work are set out in our licence referred to above.

Other than as permitted in any relevant BMJ Author's Self Archiving Policies, I confirm this Work has not been accepted for publication elsewhere, is not being considered for publication elsewhere and does not duplicate material already published. I confirm all authors consent to publication of this Work and authorise the granting of this licence.

For peer review only

The Psychosocial Impact of COVID-19 Pandemic on Frontline Healthcare Workers in Sierra Leone: We need to protect our healthcare workers!

Sia Morenike Tengbe [1], Ibrahim Franklyn Kamara [2], Desta B Ali [1], Fanny F Koroma [1], Stephen Sevalie [3, 4], Laura Dean [5], Sally Theobald [5]

1. Ministry of Health and Sanitation, Freetown, Sierra Leone
2. Health Security and Emergency Cluster, World Health Organization Country Office, Freetown, Sierra Leone
3. 34 Military Hospital, Wilberforce, Freetown, Sierra Leone
4. Case Management Pillar, National COVID-19 Emergency Response Centre, Freetown, Sierra Leone
5. Department of International Public Health, Liverpool School of Tropical Medicine, Liverpool, UK

Correspondence to Sia Morenike Tengbe; siamoreniketengbe@outlook.com

Keywords: Psychosocial, Mental health, COVID-19, Healthcare workers, Sierra Leone.

Word count: 4758

For peer review only

Abstract

Introduction

The COVID-19 pandemic has wide-reaching health and non-health consequences, especially on mental health and psychosocial wellbeing. Healthcare workers involved in COVID-19 patient care are particularly vulnerable to psychosocial distress due to increased pressure on healthcare systems. We explored the psychosocial experiences of frontline healthcare workers during the COVID-19 pandemic in Sierra Leone.

Methods

This qualitative study used purposive sampling to recruit 13 healthcare workers from different cadres across 5 COVID-19 designated facilities in Freetown, Sierra Leone. In-depth interviews were conducted remotely in July and August 2020, transcribed verbatim and analysed using the framework approach.

Results

This study identified three overarching themes: vulnerability, resilience, and support structures. Participants expressed vulnerability relating to the challenging work environment and lack of medications as key stressors resulting in anxiety, stress, anger, isolation, and stigmatisation. Signs of resilience with experiences drawn from the 2014 Ebola outbreak, teamwork and a sense of duty were also seen. Peer support was the main support structure with no professional psychosocial support services available to healthcare workers.

Conclusions

This is the first study to provide evidence on the psychosocial impacts of COVID-19 among frontline healthcare workers in Sierra Leone. Despite signs of resilience and coping mechanisms displayed, they also experienced adverse psychosocial outcomes. There is a need to focus on enhancing strategies such as psychosocial support for healthcare workers and those

For peer review only

that overall strengthen the health system to protect healthcare workers, promote resilience, and guide recommendations for interventions during future outbreaks.

Article Summary

Strengths and Limitations of this study

- To the best of our knowledge, this is the first study to provide qualitative evidence of the psychosocial impacts of COVID-19 on healthcare workers in Sierra Leone.
- Interviewed a wide range of cadre of healthcare professionals across different COVID-19 treatment centres which provided a depth of experiences and perspectives on our findings.
- Our study was conducted in 2020 during the first wave of the outbreak, healthcare workers' perceptions and experiences may have evolved over this long pandemic.
- Interviews were conducted remotely, and this may have influenced the degree to which healthcare workers may have responded.

Introduction

Globally, we are experiencing an increasing trend of infectious disease outbreaks which have had a significant impact on livelihoods and public health and safety [1]. Over the last two decades, the 2003 severe acute respiratory syndrome (SARS) outbreak, the 2009 Influenza (H1N1) pandemic, the 2012 Middle East respiratory syndrome coronavirus outbreak, the 2013–2016 Ebola virus disease epidemic in West Africa, the 2015 Zika virus disease and the ongoing COVID-19 pandemic have all had significant health and non-health impacts [2 3].

For peer review only - http://bmjopen.bmj.com/site/about/guidelines.xhtml

Healthcare workers (HCWs) are a crucial resource in the management of outbreaks and are particularly vulnerable during these periods which brings additional challenges to their already stressful work environment including the increased risk of infection. The pre-existing work stress, coupled with additional outbreak-related stressors combined, may result in psychological distress amongst the health workforce especially pronounced in frontline HCWs directly involved in the diagnosis, treatment and discharge of patients [4 5 6 7]. It is therefore essential that policies and strategies that protect frontline healthcare workers and minimise their psychosocial distress are part of an effective public outbreak response.

In Sierra Leone, during the 2014 Ebola outbreak, healthcare workers were 30 times more likely to be infected than the public [8]. This resulted in 295 healthcare worker infections and 221 deaths equating to a 6.85% loss of its total healthcare workforce [9 10]. Poor psychological outcomes including anxiety, post-traumatic stress disorder, anger, fear and community stigmatisation were reported amongst the few studies conducted during the period which focused on the outbreak's impact on healthcare workers [11 12].

Job-related stressors like increased workload, lack of personal protection equipment (PPE) and diagnostic capacity, prolonged shift hours, fear of possible infection and death of colleagues were shown to have a profound effect on healthcare workers' psychological wellbeing and coping mechanisms during the Ebola outbreak [12-14].

Since the first cases of this novel coronavirus (severe acute respiratory syndrome coronavirus 2) emerged in Wuhan, China and subsequent declaration of a pandemic on 11th March 2020, over 550 million infections and 6.3 million deaths have been reported as of July 2022 [15]. This is inclusive of more than 150,400 COVID-19 infections in healthcare workers in Africa and

For peer review only - http://bmjopen.bmj.com/site/about/guidelines.xhtml

269 in Sierra Leone [16 17]. Furthermore, a recent study in a secondary hospital in Sierra Leone shows a high rate (29%) of secondary COVID-19 infections among healthcare workers [18].

In Sierra Leone, there are growing concerns that the pandemic may trigger anxiety and post-traumatic stress among healthcare workers similar to the devastating impact of the 2014 Ebola outbreak [19]. Emerging evidence from the COVID-19 pandemic shows healthcare workers reporting high levels of psychological distress [20 21]. A systematic review and meta-analysis conducted during the first wave of the COVID-19 pandemic support this evidence with higher levels of anxiety and depression among frontline healthcare workers [22]. However, there is limited evidence from Sub-Saharan Africa focusing on the psychological impact of COVID-19 amongst healthcare workers in the region [23].

The findings of this study will provide evidence for recommendations to guide policy and practice and ensure support structures that build resilience among healthcare workers are adopted during the COVID-19 pandemic and future outbreaks. Additionally, it will also add to the global, regional, and national body of evidence on the psychosocial impact of COVID-19 on healthcare workers. The aim of the study was to explore the psychosocial experiences of frontline healthcare workers during the COVID-19 pandemic in Sierra Leone as there has been no research done to date exploring this crucial issue.

Methodology

Study setting

This qualitative study was conducted in five different coronavirus designated centres in Freetown, the capital city of Sierra Leone and the epicentre of the COVID-19 outbreak, recording the highest number of confirmed coronavirus cases [24]. During the 2014 Ebola

For peer review only - http://bmjopen.bmj.com/site/about/guidelines.xhtml

outbreak in Sierra Leone, Freetown was also one of the epicentres of the outbreak and recorded the highest prevalence (39%) of confirmed cases across the country [25].

In Sierra Leone, the management of COVID-19 patients was separated from regular health service provision to protect and maintain essential health services based on learnings from the 2014 Ebola outbreak. A total of 21 treatment centres were designated for COVID-19 across the country [26]. These treatment centres were classified into three categories based on the severity of the disease. Isolation units (suspected cases), community care centres (asymptomatic and mild cases) and COVID-19 treatment centres (moderate, severe and critical cases, and all patients over 65 years with underlying medical conditions regardless of the severity of their symptoms) [26].

Sample and recruitment

We recruited thirteen frontline healthcare workers to complete in-depth interviews across five different types of COVID-19 designated centres in Freetown. Participants were interviewed in July and August 2020. Sampling was purposive to include a range of different cadres, employment status, gender, work experience and professional roles Table 1. All participants also had prior experience providing care during the 2014 Ebola outbreak. These participants were recruited via telephone calls, emails and social media networking platforms, using our pre-established communications and connections. Two of the researchers were members of the national COVID-19 response which made it easy to identify study participants and share study information with consented participants.

Table 1: Characteristics of frontline healthcare workers that participated in the study, July-August in Freetown, Sierra Leone, 2020

For peer review only

Participants	13
Cadres	Doctors (6)<br>Nurses (6)<br>Community Health Officer (1)
Gender	Male (7)<br>Female (6)
Employment status	Government (7)<br>Military (3)<br>Volunteers (3)
Work experience	5-10 years (11)<br>>10 years (2)
Location (work site)	COVID-19 treatment centres (CTC) (6)<br>Community care centres (CCC) (3)<br>Isolation units (2)<br>National COVID-19 Emergency Response Centre (NaCOVERC) (3)

Data collection

We conducted semi-structured, one-to-one interviews exploring the psychological and social impacts of the COVID-19 pandemic on participants. The interviews were conducted by SMT remotely via Zoom or WhatsApp platforms using pretested topic guides. This ensured we collected the information within a short time, minimising disruption to the busy schedules of participants [27 28]. It was also appropriate given the COVID-19 social distancing restrictions which were in place [29 30].

The interviews lasted for about 45-60 minutes and were done in Krio (local dialect) and English (depending on participant preference) using a topic guide. All participants were given a participant information sheet a week before the interviews and encouraged to ask questions. Written informed consent was obtained prior to interviews and a demographics form was completed by all participants. We audio-recorded interviews with participants' consent, and

For peer review only - http://bmjopen.bmj.com/site/about/guidelines.xhtml

recordings were transcribed. The audio recording of interviews, interview notes and available transcripts enabled repeated revisiting of data to remain immersed in the data and to participants' original accounts, helping to enhance the validity of the results.

As this was a rapid study to provide evidence to support the ongoing COVID-19 response, interviews ceased when data saturation was reached as a commonality between themes emerged and the lead author identified no new themes emerging within the interviews.

Patient and public involvement

The study participants or the public were not involved in the design and conduct of the study. However, preliminary findings and recommendations have been shared with the case management pillar of the National COVID-19 Emergency Response centre and the Ministry of Health and Sanitation. Recommendations are designed to support improving the development of policy and practice to ensure healthcare workers are provided with psychosocial support during the pandemic and future disease outbreaks. The findings will be shared with the wider public and on social media.

Data Analysis

We used the framework approach for data analysis as it supports the generation of themes and provided clear systematic steps while maintaining the transparency of the analytic process [31]. A coding framework was developed both deductively from the topic guide and study objectives, and inductively from themes emerging from the data. This coding framework was applied to each transcript with charts developed for each theme, NVivo Version 12 was used to aid the analysis. The study authors agreed on the final themes and included rich and verbatim descriptions of participants' experiences to support these findings

For peer review only

For peer review only - http://bmjopen.bmj.com/site/about/guidelines.xhtml

Results

The result is structured into three (3) key interlinking themes- vulnerability, resilience and support systems, each with several sub-themes Table 2.

Table 2: Themes and sub-themes of the psychosocial impacts of COVID-19 on frontline healthcare workers in Freetown, Sierra Leone, 2020.

THEME	SUB-THEME
1. Vulnerability	1.1. Work environment<br>1.2. Lack of resources and medication<br>1.3. Stigmatisation<br>1.4. PTSD
2. Resilience	2.1. Essential healthcare service provision<br>2.2. Teamwork<br>2.3. Training<br>2.4. Sense of purpose/duty<br>2.5. 2014 Ebola outbreak
3. Support Systems	3.1. Peer support<br>3.2. Donations<br>3.3. Professional support<br>3.4. Lack of recognition<br>3.5. Challenges in the health system

1. Vulnerability

1.1. Work environment

"Working under stressful conditions is normal in Sierra Leone…" (HCW 08, Doctor, Male)

For peer review only - http://bmjopen.bmj.com/site/about/guidelines.xhtml

Despite acknowledging that healthcare workers are used to working under stressful conditions, increased experiences of stress, anxiety and isolation were expressed during COVID-19 patient care. These experiences peaked during the first three months of the coronavirus pandemic and seemed to be more vividly expressed by females and nurses than by males and doctors.

"When the first cases came, we were not enough at all…that time it was hell, because we had 30 beds and 4 nurses cannot deal with 30 patients..." (HCW 01, Nurse, Female)

These experiences were predominantly associated with disruptions in the work environment with increased workload, inadequate human resources, and a rapid influx of COVID-19 patients especially the critically ill.

The fear of possible infection due to patient care also fed into healthcare workers' vulnerability with concerns for personal safety and onward transmission to family members and friends.

"When a colleague became infected, it was really stressful…we did not know where she got the contact…we were quarantined, samples collected…we were scared...what if our samples turn out positive? what will we do? ...we have put our lives and families at high risk..." (HCW 06, Nurse, Female).

1.2. Lack of resources and medications

Doctors and in-charge nurses with more managerial roles expressed anger, guilt, helplessness, and frustration related to the medical commodities such as medications, oxygen cylinders and personal protective equipment at COVID-19 treatment centres.

For peer review only - http://bmjopen.bmj.com/site/about/guidelines.xhtml

"The first death in the centre was diabetic and insulin was unavailable in the unit… it is challenging when you do not have what you need at the right time…. knowing at the back of your mind that if something goes wrong, you are to blame so it was very stressful."(HCW 08, Doctor, Male).

"We faced constraints with oxygen supply and if you have three patients in need it is difficult to know how to share it…I was monitoring a patient when I saw the oxygen cylinder was almost empty so I called and was told there was no gas…I was still on the call when the patient died… I just stood there…I felt guilty that there was something that could have been done…so it affected me for a few days…it ate at my conscience…" (HCW 01, Nurse, Female)

1.3. Stigmatisation

All 13 frontline HCWs interviewed reported experiencing some form of stigmatisation either from colleagues not involved in the response or from the community. These resulting feelings of isolation and depression were equally expressed across all cadres and gender.

Our colleagues were scared for us, knowing that being at the frontline, we were at risk of becoming infected…in fact, they were also afraid of us, anytime we went to the regular hospital setting, they told us not to come close to them…" (HCW 02, Nurse, Female)

For peer review only - http://bmjopen.bmj.com/site/about/guidelines.xhtml

"When COVID-19 started, my family told me not to come home…they said I should not have joined the response…so I packed my things and went to stay in the hospital. I was in the hospital for over 1 month before I came home." (HCW 05, Nurse, Male)

1.4. Post-traumatic stress disorder symptoms

These findings also highlighted that the negative experiences from the 2014 Ebola outbreak were triggered and led to some of the psychosocial outcomes that frontline healthcare workers faced during the COVID-19 response. This was reflected in the inappropriate use of PPE despite limited supplies.

"The Ebola experience brought up some PTSD…when this corona started we observed that health workers were using too many PPE…because that was what they did during the Ebola and the fear remembering that colleagues died from Ebola…" (HCW 11, Doctor, Male)

2. Resilience

Despite the challenges, pressures and vulnerabilities, frontline healthcare workers also demonstrated resilience, growth, pride, and job satisfaction during this period.

2.1. Essential healthcare services provision

The COVID-19 pandemic caused a significant shock to the health system and despite the challenges in the work environment and shortage of medications and commodities, medical doctors mainly viewed the COVID-19 response as a success as health services adapted, were maintained and did not collapse.

For peer review only - http://bmjopen.bmj.com/site/about/guidelines.xhtml

"In terms of successes, the health system has not collapsed…. We quickly move from 30-bed, decentralised, and have treatment facilities in almost every district…. I think we have 820 beds…. our surveillance is also good at pursuing contact…" (HCW 09, Doctor, Male)

"Sometimes we get shortage of PPE, but they fast track the process, so we have been able to maintain adequate PPE supplies, we have not reached the stage to recycle PPE…this has helped us to feel safe…" (HCW 04, Doctor, Male)

2.2. Teamwork

Working long, socially isolating, and tedious hours during the early months of the pandemic resulted in a close-knit group of healthcare workers across the different COVID-19 treatment centres. The resulting sense of camaraderie and teamwork was seen as a key factor for healthcare workers to continue to provide healthcare services.

"We watch each other's back and help each other with tasks…this is one of the biggest successes we have seen during the period." (HCW 04, Doctor, Male)

Inexperienced healthcare workers, especially those recruited from other wards, were most appreciative of the close collaboration with senior colleagues as this improved their confidence.

"*When I joined the treatment centre from another unit, I found it difficult to adapt... we had a lot of patients, and it was not easy…but our senior colleagues taught and supported us …. this really help me, and I became less afraid..." (HCW 03, Nurse, Female)*

For peer review only

The healthcare workers also viewed collaborative and technical skills gained during COVID-19 patient care as making them more equipped and better prepared for future work.

"It is challenging but also a learning process as I have to deal with people from different sectors and backgrounds, not just the ones normally involved in the healthcare system..." (HCW 12, Doctor, Male)

"I have gained a lot of experience because I did not know how to use oxygen concentrators... but I can..." (HCW 03, Nurse, Female)

2.3. Training

Healthcare workers reported training centred around infection prevention and control (IPC) including the use of PPE and case management was the primary form of preparation for COVID-19 patient care. Training sessions were organised before the country's first COVID-19 case was reported on 31st March 2020 and this reduced anxiety over possible personal safety and healthcare worker infections. These training(s) also built on experiences gained during the 2014 Ebola outbreak which strengthened HCWs' competencies in IPC.

"We did refreshers trainings on how to put on PPE, IPC and hand hygiene before the COVID outbreak came into the country….so when COVID came we were not surprised…" (HCW 12, Doctor, Male)

"I think the Ebola gave us a footing especially with IPC…so it was not new…I think this is less stressful compared to the Ebola Era." (HCW 07, CHO, Female).

For peer review only

For peer review only - http://bmjopen.bmj.com/site/about/guidelines.xhtml

2.4. Sense of duty

All participants reported reminding themselves of their Hippocratic oath and the nature of their duties as a significant way of dealing with stress. Empathy towards the COVID-19 patients was also a strong motivator to continue patient care during the stressful period, with healthcare workers feeling unable to stay away and stop working despite the challenges and psychosocial outcomes.

"When I feel weak and don't want to go work I remember my nurse's and military pledge so I need to fulfil my duty…I can't stay home because you feel that it's your mom or daddy that is in the hospital bed, you need to go help…" (HCW 03, Nurse, Female)

2.5. 2014 Ebola outbreak

All the healthcare workers expressed that experiences gained while working during the 2014 Ebola outbreak were invaluable as they were more confident, better prepared psychologically and had transferable skills and competencies which reduced their levels of fear for providing care during the COVID-19 pandemic. Most participants reported feeling more afraid and stressed during the Ebola outbreak as compared to the COVID-19 pandemic demonstrating significant resilience, despite the presence of emotional triggers as previously described.

"The emotions during the Ebola was scarier than the COVID because that was the first outbreak exposure, COVID is my second so I did not have much fear to join the response because I had previous experience…" (HCW 13, Doctor, Male)

For peer review only

For peer review only - http://bmjopen.bmj.com/site/about/guidelines.xhtml

"The experience I got during Ebola was greater than this because during Ebola people were dying almost every minute…you needed great courage to work…we wore diapers inside the PPE, because if you left the ward you would not want to come back in…" (HCW 05, Nurse, Male)

3. Support structure

Frontline healthcare workers expressed vulnerability and resilience were influenced by their support structures.

3.1. Peer support

Gestures such as sharing meals, discussions before and after shifts as well as sharing jokes were expressed as the most common form of support. Several nurses expressed that encouraging words from doctors in charge of the COVID-19 unit also served as a boost. A WhatsApp group was used by some of the frontline healthcare workers to express their feelings, seek guidance and foster bonding among peers.

"We have this forum that everybody can vent out through audio clips, the experience of the day so we support each other and give advice on how to cope with the stress…" (HCW 10, Nurse, Female)

"When I feel weak…I listen to gospel songs…when I do this I feel Ok within myself…God gives me the energy and strength to be able to cope with the work." (HCW 06, Nurse, Female)

3.2. Donations

For peer review only - http://bmjopen.bmj.com/site/about/guidelines.xhtml

Donations from organisations and the community to COVID-19 treatment centres came in as medications, equipment and personal items for the COVID-19 patients, with a few targeting the healthcare workers. These gestures were received as a welcoming boost improving both patient care and morale among the staff.

"The day we received donations and had enough medications and some of the equipment we needed in the treatment centre…it lifted the mood of the entire staff, people were willing to work, absenteeism reduced…that was a big game changer." (HCW 12, Doctor, Male)

3.3. Professional support

Although there were psychosocial support services for patients in the treatment centres, this service is not accessible to frontline healthcare workers involved in the treatment of admitted COVID-19 patients. This was met with frustration from being excluded from such services by some healthcare workers.

"We have gone through the Ebola, mudslide, flooding now COVID, I know Sierra Leonean we are resilient, but we should not take it for granted…we are traumatised and full of anxiety and nobody cares about our emotional stress…" (HCW 10, Nurse, Female)

3.4. Lack of recognition

Frontline healthcare workers also reported feeling that their hard work was not adequately recognised, and appreciated by the government, especially the Ministry of Health and Sanitation. The lack of visitation by top government officials to COVID-19 treatment centres was mentioned by four healthcare workers as particularly discouraging.

For peer review only

"When the good comes, you hardly hear about the nurses… instead it is the big guns that are boasting.... what about the health workers that are in the fight…It's like nobody even cares…the minister has not even come around…those little things count." (HCW 10, Nurse, Female)

"A certificate of recognition should also be given to health workers in the frontline…this will help show that our efforts are valued and appreciated…" (HCW 13, Doctor, Male)

3.5. Health system challenges

Some participants expressed a sense of missed opportunity to capitalise on the country's history of outbreaks to strengthen its health system.

"During Ebola we had support from all over the world but with COVID-19, everyone has been grounded in their respective countries…this is a way of telling us to strengthen our health sector…" (HCW 13, Doctor, Male)

Improving conditions of service, availability of essential medications and supplies, the recruitment of volunteer healthcare workers not on the government payroll and the provision of health insurance for healthcare workers were repeatedly expressed.

"I think if we have the psychosocial counselling structure backed up by hospitals having the basics…this would help healthcare workers during outbreaks…" (HCW,11, Doctor, Male)

Discussion

For peer review only - http://bmjopen.bmj.com/site/about/guidelines.xhtml

This qualitative study explored the psychosocial experiences of frontline healthcare workers delivering COVID-19 care in Sierra Leone. The findings presented in this study are a particularly valuable in-depth qualitative analysis of psychosocial distress and extreme resilience among HCWs in the COVID-19 response. To date, the experiences of frontline healthcare workers who have been involved in both the 2014 West Africa Ebola outbreak and the COVID-19 pandemic are largely absent within the debate on psychosocial distress during epidemics. We present our conceptual framework Figure 1, to show that frontline healthcare workers' psychosocial outcomes in Sierra Leone during the COVID-19 outbreak, are shaped by their vulnerabilities and resilience, with these experiences inexplicably linked to the strengths and weaknesses of the health system.

{INSET FIGURE 1}

Similar to other COVID-19 studies, our study also shows that stress, anxiety, fatigue, isolation, and fear of infection affected frontline HCWs the most during the start of the pandemic [32 33]. This is not surprising given the novelty of the disease and the traumatic nature of infectious disease outbreaks. We would therefore recommend that psychosocial support services be implemented early to promote resilience and protect healthcare workers.

Furthermore, studies done in sub -Saharan Africa, Asia and Europe during the COVID-19 pandemic emphasised similar themes to those we identified that the lack of medications, medical equipment and oxygen supply introduced further constraints in the workplace, affecting healthcare workers' ability to carry out their duties, frequently resulting in psychological distress [20 34 35]. The shortage of PPE was highlighted in systematic reviews as a major predisposing factor for negative psychological impact on healthcare workers during the

For peer review only

COVID-19 pandemic [7 36]. However, our findings contradict this trend as HCWs did not express significant concerns in terms of the availability of PPE and were proud that the healthcare systems did not collapse. Despite this, the importance of PPE to healthcare workers in managing their fears and vulnerabilities was apparent and working with healthcare workers to achieve appropriate use of PPE based on disease conditions would support in ensuring efficient use of scarce health system resources. We recommend that investments in health system strengthening efforts should be prioritised as this is crucial in outbreak response to ensure the effectiveness of healthcare workers, improve safety and reduce stress.

Although all participants expressed psychological experiences, there were variations in the perceived levels based on gender, cadre and work experience. This study confirms findings from Ghana and the United Kingdom which highlighted that nurses are more vulnerable given their contact time with patients and in China which states that inexperienced healthcare workers express more stress while trying to adapt to the new settings [37-39]. Further explanation for the gender-related differences in our study may be because most of the female participants are nurses and males are doctors. Reflecting on this finding and considering gendered occupational segregation in the health workforce in Sierra Leone, with female nurses accounting for 90% of all nursing personnel [40]; gender considerations must be prioritised when planning psychosocial support services for healthcare workers, together with regular training and simulation exercises in emergency response to allay fear and anxiety among inexperienced healthcare workers during future outbreaks [21 24 33].

Our study adds to the overall body of evidence emerging from other studies on healthcare workers during outbreaks that direct involvement with patients had a varying effect on healthcare workers' relationships often resulting in stigmatisation increasing their

For peer review only - http://bmjopen.bmj.com/site/about/guidelines.xhtml
For peer review only

psychological distress [3 12 32]. This signifies the need for the early introduction of risk communication and community engagement programs during outbreaks to increase awareness and reduce stigma.

Resilience among healthcare workers during the COVID-19 pandemic is well documented in the literature [34 41]. In our study, however, signs of resilience were mainly because of the legacy of the 2014 Ebola outbreak, a finding which is likely transferable to a limited number of contexts. The experience gained from patient care during the Ebola outbreak was seen as training in outbreak response and formed part of healthcare workers' coping strategy. Our finding further showed that healthcare workers expressed optimism that the foundations laid by the Ebola outbreak have been built upon by the COVID-19 pandemic. Hence, they possess the necessary skills and expertise to face future outbreaks. This growing theme of resilience is also shown in the findings of a recent study to assess the impact of the COVID-19 pandemic on hospital utilisation in Sierra Leone [30]. It is worth acknowledging that despite the different nature and trajectory of both outbreaks with varying responses, there are common issues that inevitably shape the psychosocial outcomes of healthcare workers.

Measures such as training and availability of infection prevention and control measures have been found to have a pivotal impact in reducing frontline HCWs' risk of infections and a positive effect on their psychosocial outlook [3 4]. Findings from our study resonate with this body of evidence as multiple trainings were conducted for healthcare workers and the management of COVID-19 patients was separated from general care. As a result, participants felt safer, less anxious, and more confident to provide patient care. Moving forward, training a critical mass of healthcare workers that possess the prerequisite knowledge, skills and

For peer review only

competencies to provide quality healthcare to infectious disease patients that are admitted to a designated treatment centre could prove invaluable.

In line with previous studies done to assess motivators that kept HCWs working during outbreaks, we showed that the pandemic presented an opportunity for inexperienced frontline healthcare to learn new skills and develop stronger teams. Professional ethics, empathy, religion and community donations helped healthcare workers in coping with emotional struggles and physical exhaustion and focus on performing their duty [34 35 42].

Broader literature on support structures used by healthcare workers during outbreaks showed that HCWs discussed challenges and stresses with colleagues at the end of the shift and on social media platforms [4]. Our findings are in concordance with this peer support theme. However, the unavailability of counselling and professional support services for HCWs in our study resonates with findings from studies done in South Africa and Ghana but contrasts with those done in Europe where formal psychosocial support services were provided for healthcare workers [20 33 38]. This is disconcerting even with signs of resilience and success. It signifies a missed opportunity and gap presenting further evidence for the need to instil measures in place to acknowledge and protect healthcare workers from undue exposure and adverse psychosocial outcomes during crises.

Psychosocial support structures must also go together with activities that strengthen the healthcare facilities given that the work environment is a strong predisposing factor for psychosocial distress. Furthermore, improvement of the conditions of service for healthcare workers with the introduction of health insurance schemes, employment of volunteering healthcare workers, and formal recognition of service and appraisal systems for frontline

For peer review only - http://bmjopen.bmj.com/site/about/guidelines.xhtml

healthcare workers with exemplary performance during outbreak response would boost morale as well as reinforce positive conduct.

Strengths and Limitations

To the best of our knowledge, this is the first study to provide qualitative evidence of the psychosocial impacts of COVID-19 on healthcare workers in Sierra Leone. We interviewed a wide range of cadre of healthcare professionals across different COVID-19 treatment centres which provided a depth of experiences and perspectives on our findings. The lead researcher (SMT) is a Sierra Leonean medical doctor and public health professional with established rapport, and connections and understands the local situation ensuring the trustworthiness of our findings.

There were some limitations. Our study was conducted in 2020 during the first wave of the outbreak, healthcare workers' perceptions and experiences may have evolved over this long pandemic. However, this study captured the experiences of healthcare workers at the height of the pandemic. Furthermore, recently published studies have highlighted similar experiences of healthcare workers that are involved in COVID-19 patient care.

The interviews were conducted remotely, and this may have influenced the degree to which healthcare workers may have responded. However, prior established rapport and communications with participants on social media and through emails before the interview process helped to circumvent this potential challenge. This is in keeping with recommendations from other qualitative studies that used remote interviewing [43 44]. This also allowed us to

For peer review only - http://bmjopen.bmj.com/site/about/guidelines.xhtml

conduct interviews at the participant's preferred time, minimised distraction and was appropriate given the strict COVID-19 restrictions in place.

Conclusion

Our study is the first to explore the psychosocial impacts of COVID-19 among frontline healthcare workers in Sierra Leone. These different cadres of frontline healthcare workers experienced a wide range of psychosocial outcomes including anxiety, stress, guilt, anger, empathy, stigmatisation, and a sense of duty. Strong signs of resilience were also expressed due to the legacy of the 2014 Ebola outbreak. We, therefore, recommend that strategies such as formal psychosocial support for healthcare workers, improved healthcare supply chain and the training of a critical mass of healthcare workers on outbreak preparedness and response be developed and implemented urgently to promote resilience and strengthen the health system in preparation for future epidemics.

Declarations

Acknowledgements

The authors would like to thank those who gave their time to participate and contribute to the study.

Contributors

SMT conceived the initial study and IFK, ST and LD contributed to study design and ethical approval process. SMT was responsible for data collection and was assisted by IFK, DBA, FFK and SS during this stage, SMT conducted data analysis alongside ST and LD. SMT produced the original draft of the manuscript. All authors critically reviewed and edited and

For peer review only

For peer review only - http://bmjopen.bmj.com/site/about/guidelines.xhtml

provided comments on the first and subsequent drafts. All authors approved the final manuscript for submission.

Funding

This work was supported by the UK Research and Innovation (UKRI). The GCRF Accountability for Informal Urban Equity Hub ("ARISE") is a UKRI Collective Fund award with award reference ES/S00811X/1.

Competing interests

None declared

Patient consent for publication

Not required

Ethics approval

The study was reviewed and approved by the Sierra Leone Ethics and Scientific Review Committee (24072020). Informed consent was obtained from all participants.

Data sharing statement

All data relevant to the study are included in the article.

Reference

1. Bloom DE, Cadarette D. Infectious Disease Threats in the Twenty-First Century: Strengthening the Global Response. *Frontiers in Immunology* 2019;10 doi: 10.3389/fimmu.2019.00549
2. Baker RE, Mahmud AS, Miller IF, et al. Infectious disease in an era of global change. *Nature Reviews Microbiology* 2022;20(4):193-205. doi: 10.1038/s41579-021-00639-z

For peer review only - http://bmjopen.bmj.com/site/about/guidelines.xhtml

3. Kisely S, Warren N, McMahon L, et al. Occurrence, prevention, and management of the psychological effects of emerging virus outbreaks on healthcare workers: rapid review and meta-analysis. *BMJ* 2020;369:m1642. doi: 10.1136/bmj.m1642
4. Billings J, Ching BCF, Gkofa V, et al. Experiences of frontline healthcare workers and their views about support during COVID-19 and previous pandemics: a systematic review and qualitative meta-synthesis. *BMC Health Services Research* 2021;21(1):923. doi: 10.1186/s12913-021-06917-z
5. Adams JG, Walls RM. Supporting the Health Care Workforce During the COVID-19 Global Epidemic. *Jama* 2020 doi: 10.1001/jama.2020.3972 [published Online First: 2020/03/13]
6. Lai J, Ma S, Wang Y, et al. Factors Associated With Mental Health Outcomes Among Health Care Workers Exposed to Coronavirus Disease 2019. *JAMA Netw Open* 2020;3(3):e203976. doi: 10.1001/jamanetworkopen.2020.3976 [published Online First: 2020/03/24]
7. Bekele F, Hajure M. Magnitude and determinants of the psychological impact of COVID-19 among health care workers: A systematic review. *SAGE Open Medicine* 2021;9:20503121211012512. doi: 10.1177/20503121211012512
8. Nuzzo JB, Meyer D, Snyder M, et al. What makes health systems resilient against infectious disease outbreaks and natural hazards? Results from a scoping review. *BMC Public Health* 2019;19 doi: 10.1186/s12889-019-7707-z
9. GOSL. National Ebola Recovery Strategy for Sierra Leone, 2015.
10. Evans DK, Goldstein M, Popova A. Health-care worker mortality and the legacy of the Ebola epidemic. *Lancet Glob Health* 2015;3(8):e439-e40. doi: 10.1016/s2214-109x(15)00065-0 [published Online First: 2015/07/15]
11. Shultz JM, Baingana F, Neria Y. The 2014 Ebola outbreak and mental health: current status and recommended response. *Jama* 2015;313(6):567-8. doi: 10.1001/jama.2014.17934 [published Online First: 2014/12/23]
12. Raven J, Wurie H, Witter S. Health workers' experiences of coping with the Ebola epidemic in Sierra Leone's health system: a qualitative study. *BMC Health Services Research* 2018;18(1):1-9. doi: doi:10.1186/s12913-018-3072-3
13. McMahon SA, Ho LS, Brown H, et al. Healthcare providers on the frontlines: a qualitative investigation of the social and emotional impact of delivering health services during Sierra Leone's Ebola epidemic. *Health Policy Plan* 2016;31(9):1232-9. doi: 10.1093/heapol/czw055 [published Online First: 2016/06/10]
14. Wurie H, Witter S, Raven J. 'Fighting a battle': Ebola, health workers and the health system in Sierra Leone. 2016
15. World Health Organization. WHO Coronavirus (COVID-19) Dashboard 2022 [Available from: https://covid19.who.int/ accessed 11 May 2022 2022.
16. World Health Organization. Only 1 in 4 African health workers fully vaccinated against COVID-19 2021 [Available from: https://www.afro.who.int/news/only-1-4-african-health-workers-fully-vaccinated-against-covid-19 accessed 12 May 2021.
17. World Health Organization. Update on COVID-19 Sierra Leone 2022 [Available from: https://who.maps.arcgis.com/apps/dashboards/e5d453431c2143639d2ea3ef585e20a6.
18. Squire JS, Dadzie D, Nyarko KM, et al. Risk Factors for COVID-19 infection among Hospital Healthcare Workers, Sierra Leone, 2020. *Journal of Interventional Epidemiology and Public Health* 2022;5(4) doi: doi:10.11604/JIEPH.supp.2022.5.1.1198
19. Dean L, Cooper J, Wurie H, et al. Psychological resilience, fragility and the health workforce: lessons on pandemic preparedness from Liberia and Sierra Leone. *BMJ global health* 2020;5(9):e002873. doi: 10.1136/bmjgh-2020-002873

For peer review only - http://bmjopen.bmj.com/site/about/guidelines.xhtml

20. Aughterson H, McKinlay AR, Fancourt D, et al. Psychosocial impact on frontline health and social care professionals in the UK during the COVID-19 pandemic: a qualitative interview study. *BMJ Open* 2021;11(2):e047353. doi: 10.1136/bmjopen-2020-047353
21. Daneshvar E, Otterbach S, Alameddine M, et al. Sources of anxiety among health care workers in Tehran during the COVID-19 pandemic. *Health Policy and Planning* 2022;37(3):310-21. doi: 10.1093/heapol/czab136
22. Sun P, Wang M, Song T, et al. The Psychological Impact of COVID-19 Pandemic on Health Care Workers: A Systematic Review and Meta-Analysis. *Frontiers in Psychology* 2021;12 doi: 10.3389/fpsyg.2021.626547
23. Tessema GA, Kinfu Y, Dachew BA, et al. The COVID-19 pandemic and healthcare systems in Africa: a scoping review of preparedness, impact and response. *BMJ Global Health* 2021;6(12):e007179. doi: 10.1136/bmjgh-2021-007179
24. Kamara IF, Tengbe SM, Fofanah BD, et al. Infection Prevention and Control in Three Tertiary Healthcare Facilities in Freetown, Sierra Leone during the COVID-19 Pandemic: More Needs to Be Done! *Int J Environ Res Public Health* 2022;19(9) doi: 10.3390/ijerph19095275 [published Online First: 2022/05/15]
25. Dietz PM, Centers for Disease Control and Prevention Ebola Response Group A, Georgia, Jambai A, et al. Epidemiology and Risk Factors for Ebola Virus Disease in Sierra Leone—23 May 2014 to 31 January 2015. *Clinical Infectious Diseases* 2015;61(11):1648-54. doi: 10.1093/cid/civ568
26. Kamara IF, Kumar AMV, Maruta A, et al. Antibiotic Use in Suspected and Confirmed COVID-19 Patients Admitted to Health Facilities in Sierra Leone in 2020-2021: Practice Does Not Follow Policy. *Int J Environ Res Public Health* 2022;19(7) doi: 10.3390/ijerph19074005 [published Online First: 2022/04/13]
27. DiCicco-Bloom B, Crabtree BF. The qualitative research interview. *Medical Education* 2006;40(4):314-21. doi: 10.1111/j.1365-2929.2006.02418.x
28. DeJonckheere M, Vaughn LM. Semistructured interviewing in primary care research: a balance of relationship and rigour. 2019 doi: 10.1136/fmch-2018-000057
29. Thompson A. Why Most of Africa's Data is Used on WhatsApp 2018 [Available from: https://theculturetrip.com/africa/articles/why-most-of-africas-data-is-used-on-whatsapp/ accessed 12 April 2020.
30. Sevalie S, Youkee D, van Duinen AJ, et al. The impact of the COVID-19 pandemic on hospital utilisation in Sierra Leone. *BMJ Global Health* 2021;6(10):e005988. doi: 10.1136/bmjgh-2021-005988
31. Gale NK, Heath G, Cameron E, et al. Using the framework method for the analysis of qualitative data in multi-disciplinary health research. *BMC Med Res Methodol* 2013;13:117. doi: 10.1186/1471-2288-13-117
32. Sirois FM, Owens J. Factors Associated With Psychological Distress in Health-Care Workers During an Infectious Disease Outbreak: A Rapid Systematic Review of the Evidence. *Frontiers in Psychiatry* 2021;11 doi: 10.3389/fpsyt.2020.589545
33. Dawood B, Tomita A, Ramlall S. 'Unheard,' 'uncared for' and 'unsupported': The mental health impact of Covid -19 on healthcare workers in KwaZulu-Natal Province, South Africa. *PLOS ONE* 2022;17(5):e0266008. doi: 10.1371/journal.pone.0266008
34. Shah J, Monroe-Wise A, Talib Z, et al. Mental health disorders among healthcare workers during the COVID-19 pandemic: a cross-sectional survey from three major hospitals in Kenya. *BMJ Open* 2021;11(6):e050316. doi: 10.1136/bmjopen-2021-050316
35. De Kock JH, Latham HA, Leslie SJ, et al. A rapid review of the impact of COVID-19 on the mental health of healthcare workers: implications for supporting psychological well-being. *BMC Public Health* 2021;21(1):104. doi: 10.1186/s12889-020-10070-3

For peer review only - http://bmjopen.bmj.com/site/about/guidelines.xhtml

36. Pappa S, Ntella V, Giannakas T, et al. Prevalence of depression, anxiety, and insomnia among healthcare workers during the COVID-19 pandemic: A systematic review and meta-analysis. *Brain Behav Immun* 2020 doi: 10.1016/j.bbi.2020.05.026
37. Liu Q, Luo D, Haase JE, et al. The experiences of health-care providers during the COVID-19 crisis in China: a qualitative study. *The Lancet Global Health* 2020;8(6):e790-e98. doi: 10.1016/S2214-109X(20)30204-7
38. Ofori AA, Osarfo J, Agbeno EK, et al. Psychological impact of COVID-19 on health workers in Ghana: A multicentre, cross-sectional study. *SAGE Open Medicine* 2021;9:20503121211000919. doi: 10.1177/20503121211000919
39. Lamb D, Gnanapragasam S, Greenberg N, et al. Psychosocial impact of the COVID-19 pandemic on 4378 UK healthcare workers and ancillary staff: initial baseline data from a cohort study collected during the first wave of the pandemic. *Occupational and Environmental Medicine* 2021;78(11):801. doi: 10.1136/oemed-2020-107276
40. World Health Organization. Sex distribution of health workers 2022 [Available from: https://apps.who.int/gho/data/node.main.HWFGRP_BYSEX?lang=en accessed 5 June 2022.
41. Kirabira J, Ben Forry J, Ssebuufu R, et al. Psychological distress and associated factors among hospital workers in Uganda during the COVID-19 lockdown – A multicentre study. *Heliyon* 2022;8(1):e08807. doi: https://doi.org/10.1016/j.heliyon.2022.e08807
42. Kisely S, Warren N, McMahon L, et al. Occurrence, prevention, and management of the psychological effects of emerging virus outbreaks on healthcare workers: rapid review and meta-analysis. 2020 doi: 10.1136/bmj.m1642
43. Deakin H, Wakefield K. Skype interviewing: reflections of two PhD researchers:. http://dxdoiorg/101177/1468794113488126 2013 doi: 10.1177_1468794113488126
44. Johnson DR, Scheitle CP, Ecklund EH. Beyond the In-Person Interview? How Interview Quality Varies Across In-person, Telephone, and Skype Interviews. *Social Science Computer Review* 2019;39(6):1142-58. doi: 10.1177/0894439319893612

For peer review only - http://bmjopen.bmj.com/site/about/guidelines.xhtml

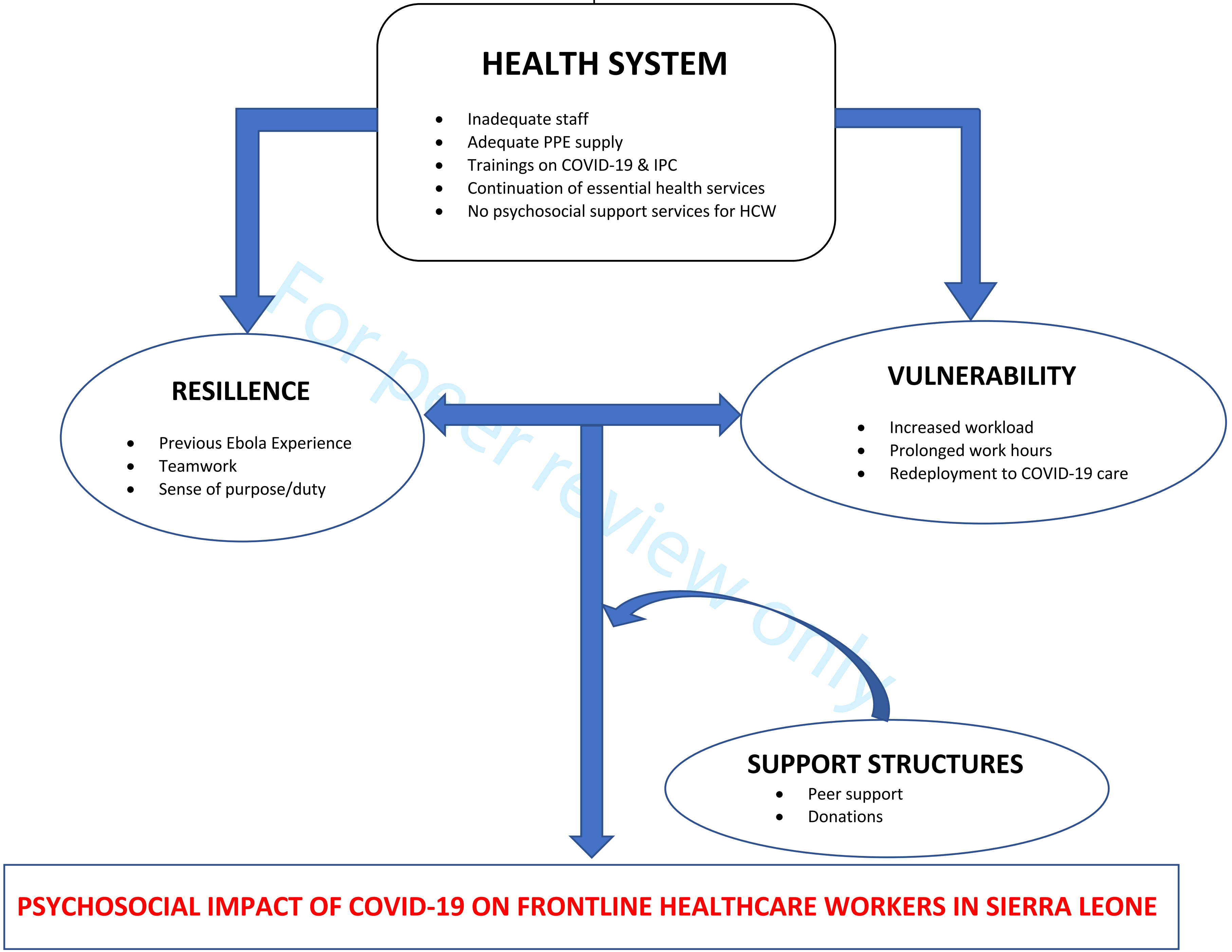
HEALTH SYSTEM
Inadequate staff
Adequate PPE supply
Trainings on COVID-19 & IPC
Continuation of essential health services
No psychosocial support services for HCW
RESILLENCE
Previous Ebola Experience
Teamwork
Sense of purpose/duty
VULNERABILITY
Increased workload
Prolonged work hours
Redeployment to COVID-19 care
SUPPORT STRUCTURES
Peer support
Donations
PSYCHOSOCIAL IMPACT OF COVID-19 ON FRONTLINE HEALTHCARE WORKERS IN SIERRA LEONE

COREQ (COnsolidated criteria for REporting Qualitative research) Checklist

A checklist of items that should be included in reports of qualitative research. You must report the page number in your manuscript where you consider each of the items listed in this checklist. If you have not included this information, either revise your manuscript accordingly before submitting or note N/A.

Topic	Item No.	Guide Questions/Description	Reported on Page No.
**Domain 1: Research team and reflexivity**			
Personal characteristics			
Interviewer/facilitator	1	Which author/s conducted the interview or focus group?	
Credentials	2	What were the researcher's credentials? E.g. PhD, MD	
Occupation	3	What was their occupation at the time of the study?	
Gender	4	Was the researcher male or female?	
Experience and training	5	What experience or training did the researcher have?	
Relationship with participants			
Relationship established	6	Was a relationship established prior to study commencement?	
Participant knowledge of the interviewer	7	What did the participants know about the researcher? e.g. personal goals, reasons for doing the research	
Interviewer characteristics	8	What characteristics were reported about the inter viewer/facilitator? e.g. Bias, assumptions, reasons and interests in the research topic	
**Domain 2: Study design**			
Theoretical framework			
Methodological orientation and Theory	9	What methodological orientation was stated to underpin the study? e.g. grounded theory, discourse analysis, ethnography, phenomenology, content analysis	
Participant selection			
Sampling	10	How were participants selected? e.g. purposive, convenience, consecutive, snowball	
Method of approach	11	How were participants approached? e.g. face-to-face, telephone, mail, email	
Sample size	12	How many participants were in the study?	
Non-participation	13	How many people refused to participate or dropped out? Reasons?	
Setting			
Setting of data collection	14	Where was the data collected? e.g. home, clinic, workplace	
Presence of non-participants	15	Was anyone else present besides the participants and researchers?	
Description of sample	16	What are the important characteristics of the sample? e.g. demographic data, date	
Data collection			
Interview guide	17	Were questions, prompts, guides provided by the authors? Was it pilot tested?	
Repeat interviews	18	Were repeat inter views carried out? If yes, how many?	
Audio/visual recording	19	Did the research use audio or visual recording to collect the data?	
Field notes	20	Were field notes made during and/or after the inter view or focus group?	
Duration	21	What was the duration of the inter views or focus group?	
Data saturation	22	Was data saturation discussed?	
Transcripts returned	23	Were transcripts returned to participants for comment and/or	

For peer review only

Topic	Item No.	Guide Questions/Description	Reported on Page No.
		correction?	
**Domain 3: analysis and findings**			
Data analysis			
Number of data coders	24	How many data coders coded the data?	
Description of the coding tree	25	Did authors provide a description of the coding tree?	
Derivation of themes	26	Were themes identified in advance or derived from the data?	
Software	27	What software, if applicable, was used to manage the data?	
Participant checking	28	Did participants provide feedback on the findings?	
Reporting			
Quotations presented	29	Were participant quotations presented to illustrate the themes/findings? Was each quotation identified? e.g. participant number	
Data and findings consistent	30	Was there consistency between the data presented and the findings?	
Clarity of major themes	31	Were major themes clearly presented in the findings?	
Clarity of minor themes	32	Is there a description of diverse cases or discussion of minor themes?	

Developed from: Tong A, Sainsbury P, Craig J. Consolidated criteria for reporting qualitative research (COREQ): a 32-item checklist for interviews and focus groups. *International Journal for Quality in Health Care*. 2007. Volume 19, Number 6: pp. 349 – 357

Once you have completed this checklist, please save a copy and upload it as part of your submission. DO NOT include this checklist as part of the main manuscript document. It must be uploaded as a separate file.

For peer review only

BMJ Open

The Psychosocial Impact of COVID-19 Pandemic on Frontline Healthcare Workers in Sierra Leone: a qualitative study

Journal:	*BMJ Open*
Manuscript ID	bmjopen-2022-068551.R1
Article Type:	Original research
Date Submitted by the Author:	10-Apr-2023
Complete List of Authors:	Tengbe, Sia Morenike; Sierra Leone Ministry of Health and Sanitation<br>Kamara, Ibrahim Franklyn; World Health Organisation Country Office for Sierra Leone, Emergency Preparedness and Response Cluster<br>Ali, Desta B; Sierra Leone Ministry of Health and Sanitation<br>Koroma, Fanny F; Sierra Leone Ministry of Health and Sanitation<br>Sevalie, Stephen; 34 Military Hospital, Wilberforce; Case Management Pillar, National COVID-19 Emergency Response Centre,<br>Dean, Laura; Liverpool School of Tropical Medicine, International Public Health<br>Theobald, Sally ; Liverpool School of Tropical Medicine, International Public Health
**Primary Subject Heading**:	Public health
Secondary Subject Heading:	Qualitative research, Mental health, Infectious diseases
Keywords:	COVID-19, Public health < INFECTIOUS DISEASES, MENTAL HEALTH

SCHOLARONE™ Manuscripts

I, the Submitting Author has the right to grant and does grant on behalf of all authors of the Work (as defined in the below author licence), an exclusive licence and/or a non-exclusive licence for contributions from authors who are: i) UK Crown employees; ii) where BMJ has agreed a CC-BY licence shall apply, and/or iii) in accordance with the terms applicable for US Federal Government officers or employees acting as part of their official duties; on a worldwide, perpetual, irrevocable, royalty-free basis to BMJ Publishing Group Ltd ("BMJ") its licensees and where the relevant Journal is co-owned by BMJ to the co-owners of the Journal, to publish the Work in this journal and any other BMJ products and to exploit all rights, as set out in our licence.

The Submitting Author accepts and understands that any supply made under these terms is made by BMJ to the Submitting Author unless you are acting as an employee on behalf of your employer or a postgraduate student of an affiliated institution which is paying any applicable article publishing charge ("APC") for Open Access articles. Where the Submitting Author wishes to make the Work available on an Open Access basis (and intends to pay the relevant APC), the terms of reuse of such Open Access shall be governed by a Creative Commons licence – details of these licences and which Creative Commons licence will apply to this Work are set out in our licence referred to above.

Other than as permitted in any relevant BMJ Author's Self Archiving Policies, I confirm this Work has not been accepted for publication elsewhere, is not being considered for publication elsewhere and does not duplicate material already published. I confirm all authors consent to publication of this Work and authorise the granting of this licence.

For peer review only

The Psychosocial Impact of COVID-19 Pandemic on Frontline Healthcare Workers in Sierra Leone: a qualitative study

Sia Morenike Tengbe [1], Ibrahim Franklyn Kamara [2], Desta B Ali [1], Fanny F Koroma [1], Stephen Sevalie [3, 4], Laura Dean [5], Sally Theobald [5]

1. Ministry of Health and Sanitation, Freetown, Sierra Leone
2. Emergency Preparedness and Response Cluster, World Health Organization Country Office, Freetown, Sierra Leone
3. 34 Military Hospital, Wilberforce, Freetown, Sierra Leone
4. Case Management Pillar, National COVID-19 Emergency Response Centre, Freetown, Sierra Leone
5. Department of International Public Health, Liverpool School of Tropical Medicine, Liverpool, UK

Correspondence to Sia Morenike Tengbe; siamoreniketengbe@outlook.com

Keywords: Psychosocial, Mental health, COVID-19, Healthcare workers, Sierra Leone.

Word count: 4895

Abstract

Introduction

The COVID-19 pandemic has wide-reaching health and non-health consequences, especially on mental health and psychosocial wellbeing. Healthcare workers involved in COVID-19 patient care are particularly vulnerable to psychosocial distress due to increased pressure on healthcare systems. We explored the psychosocial experiences of frontline healthcare workers during the COVID-19 pandemic in Sierra Leone.

Methods

This qualitative study used purposive sampling to recruit 13 healthcare workers from different cadres across five designated COVID-19 treatment centres in Freetown, Sierra Leone. In-depth interviews were conducted remotely in July and August 2020, transcribed verbatim and analysed using the framework approach.

Results

This study identified three overarching themes: vulnerability, resilience, and support structures. Participants expressed vulnerability relating to the challenging work environment and lack of medications as key stressors resulting in anxiety, stress, anger, isolation, and stigmatisation. Signs of resilience with experiences drawn from the 2014 Ebola outbreak, teamwork and a sense of duty were also seen. Peer support was the main support structure with no professional psychosocial support services available to healthcare workers.

Conclusions

This is the first study to provide evidence of the psychosocial impacts of COVID-19 among frontline healthcare workers in Sierra Leone. Despite signs of resilience and coping mechanisms displayed, they also experienced adverse psychosocial outcomes. There is a need to focus on enhancing strategies such as psychosocial support for healthcare workers and those

For peer review only

that overall strengthen the health system to protect healthcare workers, promote resilience, and guide recommendations for interventions during future outbreaks.

Article Summary

Strengths and Limitations of this study

- To the best of our knowledge, this is the first study to provide qualitative evidence of the psychosocial impacts of COVID-19 on healthcare workers in Sierra Leone.
- Interviewed different cadres of healthcare professionals across different COVID-19 treatment centres which provided a depth of experiences and perspectives on our findings.
- Our study was conducted in 2020 during the first wave of the outbreak, healthcare workers' perceptions and experiences may have evolved over this long pandemic.
- Interviews were conducted remotely, and this may have influenced the degree to which healthcare workers may have responded.

Introduction

Globally, we are experiencing an increasing trend of infectious disease outbreaks which have had a significant impact on livelihoods and public health and safety [1]. Over the last two decades, the 2003 severe acute respiratory syndrome (SARS) outbreak, the 2009 Influenza (H1N1) pandemic, the 2012 Middle East respiratory syndrome coronavirus outbreak, the 2013–2016 Ebola virus disease epidemic in West Africa, the 2015 Zika virus disease and the ongoing COVID-19 pandemic have all had significant health and non-health impacts [2 3].

For peer review only - http://bmjopen.bmj.com/site/about/guidelines.xhtml

Healthcare workers (HCWs) are a crucial resource in the management of outbreaks and are particularly vulnerable during these periods which brings additional challenges to their already stressful work environment including the increased risk of infection. The pre-existing work stress, coupled with additional outbreak-related stressors combined, may result in psychological distress amongst the health workforce especially pronounced in frontline HCWs directly involved in the diagnosis, treatment and discharge of patients [4 5 6 7]. It is therefore essential that policies and strategies that protect frontline healthcare workers and minimise their psychosocial distress are part of an effective public outbreak response.

In Sierra Leone, during the 2014 Ebola outbreak, healthcare workers were 30 times more likely to be infected than the public [8]. This resulted in 295 healthcare worker infections and 221 deaths equating to a 6.85% loss of its total healthcare workforce [9 10]. Poor psychological outcomes including anxiety, post-traumatic stress disorder, anger, fear and community stigmatisation were reported amongst the few studies conducted during the period which focused on the outbreak's impact on healthcare workers [11 12]. Despite feeling more confident and knowledgeable in providing care for Ebola survivors, negative psychological outcomes such as anxiety and anger still lingered among healthcare workers after the end of the Ebola outbreak [13]

Job-related stressors like increased workload, lack of personal protection equipment (PPE) and diagnostic capacity, prolonged shift hours, fear of possible infection and death of colleagues were shown to have a profound effect on healthcare workers' psychological wellbeing and coping mechanisms during the Ebola outbreak [12 14 15].

For peer review only - http://bmjopen.bmj.com/site/about/guidelines.xhtml

Since the first cases of this novel coronavirus (severe acute respiratory syndrome coronavirus 2) emerged in Wuhan, China and the subsequent declaration of a pandemic on 11th March 2020, over 550 million infections and 6.3 million deaths have been reported as of July 2022 [16]. This is inclusive of more than 150,400 COVID-19 infections in healthcare workers in Africa and 269 in Sierra Leone [17 18]. Furthermore, a recent study in a secondary hospital in Sierra Leone shows a high rate (29%) of secondary COVID-19 infections among healthcare workers [19].

In Sierra Leone, there are growing concerns that the pandemic may trigger anxiety and post-traumatic stress among healthcare workers similar to the devastating impact of the 2014 Ebola outbreak [20]. Emerging evidence from the COVID-19 pandemic shows healthcare workers reporting high levels of psychological distress [21 22]. A systematic review and meta-analysis conducted during the first wave of the COVID-19 pandemic support this evidence with higher levels of anxiety and depression among frontline healthcare workers [23]. However, there is limited evidence from Sub-Saharan Africa focusing on the psychological impact of COVID-19 amongst healthcare workers in the region [24].

The findings of this study could provide evidence for recommendations to guide policy and practice and ensure support structures that build resilience among healthcare workers are adopted during the COVID-19 pandemic and future outbreaks. Additionally, it will also add to the global, regional, and national body of evidence on the psychosocial impact of COVID-19 on healthcare workers. The aim of this qualitative study was to explore the psychosocial experiences of frontline healthcare workers during the COVID-19 pandemic in Sierra Leone as there has been no research done to date exploring this crucial issue.

Methodology

Study design

For peer review only - http://bmjopen.bmj.com/site/about/guidelines.xhtml

123 An explorative qualitative interview study with data analysed using the framework approach, which supports the generation of themes and provided clear systematic steps while maintaining the transparency of the analytic process[25]. The study is based on the COnsolidated criteria for REporting Qualitative research (COREQ) [26].

128 **Study setting**

129 This study was conducted in five out of the eight designated COVID-19 treatment centres in Freetown, the capital city of Sierra Leone and the epicentre of the COVID-19 outbreak. [27]. During the 2014 Ebola outbreak in Sierra Leone, Freetown was also one of the epicentres of the outbreak and recorded the highest prevalence (39%) of confirmed cases across the country [28].

135 In Sierra Leone, the management of COVID-19 patients was separated from regular health service provision to protect and maintain essential health services based on learnings from the 2014 Ebola outbreak. A total of 21 treatment centres were designated for COVID-19 across the country [29]. These treatment centres were classified into three categories based on the severity of the disease. Isolation units (suspected cases), community care centres (asymptomatic and mild cases) and COVID-19 treatment centres (moderate, severe and critical cases, and all patients over 65 years with underlying medical conditions regardless of the severity of their symptoms) [29].

144 **Sample and recruitment**

145 We recruited thirteen frontline healthcare workers to complete in-depth interviews across five COVID-19 designated treatment centres in Freetown. Participants were interviewed in July and August 2020. Sampling was purposive to include a range of different cadres, employment

For peer review only - http://bmjopen.bmj.com/site/about/guidelines.xhtml

statuses, gender, work experience and professional roles Table 1. All participants also had prior experience providing care during the 2014 Ebola outbreak. These participants were recruited via telephone calls, emails and social media networking platforms, using our pre-established communications and connections. Two of the researchers were members of the national COVID-19 response which made it easy to identify study participants and share study information with consented participants.

Table 1: Characteristics of frontline healthcare workers that participated in the study, July-August in Freetown, Sierra Leone, 2020

Participants	13
Cadres	Doctors (6)<br>Nurses (6)<br>Community Health Officer (1)
Gender	Male (7)<br>Female (6)
Employment status	Government (7)<br>Military (3)<br>Volunteers (3)
Work experience	5-10 years (11)<br>>10 years (2)
Location (work site)	COVID-19 treatment centres (CTC) (6)<br>Community care centres (CCC) (3)<br>Isolation units (2)<br>National COVID-19 Emergency Response Centre (NaCOVERC) (3)

Data collection

We conducted semi-structured, one-to-one interviews exploring the psychological and social impacts of the COVID-19 pandemic on participants. The interviews were conducted by SMT

For peer review only - http://bmjopen.bmj.com/site/about/guidelines.xhtml

(lead researcher) remotely via Zoom or WhatsApp platforms using a pretested topic guide. The development of the topic guide was guided by existing behavioural change theories on health stress and coping mechanisms. [30] This pretested topic guide has been provided as an online supplementary material. This ensured we collected the information within a short time, minimising disruption to the busy schedules of participants [31 32]. It was also appropriate given the COVID-19 social distancing restrictions which were in place [33 34].

The interviews lasted for about 45-60 minutes and were done in Krio (local dialect) and English (depending on participant preference) using a topic guide. All participants were given a participant information sheet a week before the interviews and encouraged to ask questions. Written informed consent was obtained prior to interviews and a demographics form was completed by all participants. We audio-recorded interviews with participants' consent, and recordings were transcribed by the lead researcher (SMT) who has experience in qualitative research. The audio recording of interviews, interview notes and available transcripts enabled repeated revisiting of data to remain immersed in the data and to participants' original accounts, helping to enhance the validity of the results.

As this was a rapid study to provide evidence to support the ongoing COVID-19 response, interviews ceased when data saturation was reached as a commonality between themes emerged and the lead author identified no new themes emerging within the interviews.

Patient and public involvement

The study participants or the public were not involved in the design and conduct of the study. However, preliminary findings and recommendations have been shared with the case management pillar of the National COVID-19 Emergency Response centre and the Ministry of Health and Sanitation. Recommendations are designed to support improving the

For peer review only

For peer review only - http://bmjopen.bmj.com/site/about/guidelines.xhtml

development of policy and practice to ensure healthcare workers are provided with psychosocial support during the pandemic and future disease outbreaks. The findings will be shared with the wider public and on social media.

Data Analysis

Following anonymisation by the lead researcher (SMT), transcripts were uploaded to NVivo Version 12 to aid analysis. Using the framework analysis approach, three researchers (SMT, ST and LD) initially read transcripts independently and discussed any emerging codes of potential significance to the research objectives. A coding framework was developed both deductively from the topic guide and study objectives, and inductively from themes emerging from the data. This coding framework was applied to each transcript with charts developed for each theme. All study authors agreed on the final themes and included rich and verbatim descriptions of participants' experiences to support these findings.

Results

The result is structured into three (3) key interlinking themes- vulnerability, resilience and support systems, each with several sub-themes Table 2.

Table 2: Themes and sub-themes of the psychosocial impacts of COVID-19 on frontline healthcare workers in Freetown, Sierra Leone, 2020.

THEME	SUB-THEME
1. Vulnerability	1.1. Work environment<br>1.2. Lack of resources and medication

For peer review only

	1.3.	Stigmatisation
	1.4.	PTSD
	1.5.	Lack of recognition
2. Resilience	2.1.	Essential healthcare service provision
	2.2.	Teamwork
	2.3.	Training
	2.4.	Sense of purpose/duty
	2.5.	2014 Ebola outbreak
3. Support Systems	3.1.	Peer support
	3.2.	Donations
	3.3.	Professional support
	3.4.	Challenges in the health system

For peer review only

1. Vulnerability

1.1. Work environment

"Working under stressful conditions is normal in Sierra Leone…" (HCW 08, Doctor, Male)

Despite acknowledging that healthcare workers are used to working under stressful conditions, increased experiences of stress, anxiety and isolation were expressed during COVID-19 patient care. These experiences peaked during the first three months of the coronavirus pandemic and seemed to be more vividly expressed by females and nurses than by males and doctors.

"When the first cases came, we were not enough at all…that time it was hell, because we had 30 beds and 4 nurses cannot deal with 30 patients..." (HCW 01, Nurse, Female)

For peer review only - http://bmjopen.bmj.com/site/about/guidelines.xhtml

Healthcare workers feeling overwhelmed were predominantly associated with disruptions in the work environment with increased workload, inadequate human resources, and a rapid influx of COVID-19 patients especially the critically ill.

The fear of possible infection due to patient care also fed into healthcare workers' vulnerability with concerns for personal safety and onward transmission to family members and friends.

"When a colleague became infected, it was really stressful…we did not know where she got the contact…we were quarantined, samples collected…we were scared...what if our samples turn out positive? what will we do? ...we have put our lives and families at high risk..." (HCW 06, Nurse, Female).

1.2. Lack of resources and medications

Doctors and in-charge nurses with more managerial roles expressed anger, guilt, helplessness, and frustration related to the medical commodities such as medications, oxygen cylinders and personal protective equipment at COVID-19 treatment centres.

"The first death in the centre was diabetic and insulin was unavailable in the unit… it is challenging when you do not have what you need at the right time…. knowing at the back of your mind that if something goes wrong, you are to blame so it was very stressful."(HCW 08, Doctor, Male).

For peer review only - http://bmjopen.bmj.com/site/about/guidelines.xhtml

"We faced constraints with oxygen supply and if you have three patients in need it is difficult to know how to share it…I was monitoring a patient when I saw the oxygen cylinder was almost empty so I called and was told there was no gas…I was still on the call when the patient died… I just stood there…I felt guilty that there was something that could have been done…so it affected me for a few days…it ate at my conscience…" (HCW 01, Nurse, Female)

1.3. Stigmatisation

Stigmatisation either from colleagues not involved in the response and from the community resulted in HCWs feeling isolated and depressed. These feelings were equally expressed across all cadres and gender.

Our colleagues were scared for us, knowing that being at the frontline, we were at risk of becoming infected…in fact, they were also afraid of us, anytime we went to the regular hospital setting, they told us not to come close to them…" (HCW 02, Nurse, Female)

"When COVID-19 started, my family told me not to come home…they said I should not have joined the response…so I packed my things and went to stay in the hospital. I was in the hospital for over 1 month before I came home." (HCW 05, Nurse, Male)

1.4. Post-traumatic stress disorder symptoms

These findings also highlighted that the negative experiences from the 2014 Ebola outbreak such as fear and anxiety were triggered and led to some of the psychosocial outcomes frontline

For peer review only - http://bmjopen.bmj.com/site/about/guidelines.xhtml

healthcare workers faced during the COVID-19 response. This was reflected in the inappropriate use of PPE despite limited supplies.

"The Ebola experience brought up some PTSD…when this corona started we observed that health workers were using too many PPE…because that was what they did during the Ebola and the fear remembering that colleagues died from Ebola…" (HCW 11, Doctor, Male)

1.5. Lack of recognition

Frontline healthcare workers also reported feeling that their hard work was not adequately recognised, and appreciated by the government, especially the Ministry of Health and Sanitation. The lack of visitation by top government officials to COVID-19 treatment centres was mentioned by four healthcare workers as particularly discouraging.

"When the good comes, you hardly hear about the nurses… instead it is the big guns that are boasting.... what about the health workers that are in the fight…It's like nobody even cares…the minister has not even come around…those little things count." (HCW 10, Nurse, Female)

"A certificate of recognition should also be given to health workers in the frontline…this will help show that our efforts are valued and appreciated…" (HCW 13, Doctor, Male)

2. Resilience

Despite the challenges, pressures and vulnerabilities, frontline healthcare workers also demonstrated resilience, growth, pride, and job satisfaction during this period.

For peer review only - http://bmjopen.bmj.com/site/about/guidelines.xhtml

2.1. Essential healthcare services provision

The COVID-19 pandemic caused a significant shock to the health system and despite the challenges in the work environment and shortage of medications and commodities, medical doctors mainly viewed the COVID-19 response as a success as health services adapted, were maintained and did not collapse.

"In terms of successes, the health system has not collapsed…. We quickly move from 30-bed, decentralised, and have treatment facilities in almost every district…. I think we have 820 beds…. our surveillance is also good at pursuing contact…" (HCW 09, Doctor, Male)

"Sometimes we get shortage of PPE, but they fast track the process, so we have been able to maintain adequate PPE supplies, we have not reached the stage to recycle PPE…this has helped us to feel safe…" (HCW 04, Doctor, Male)

2.2. Teamwork

Working long, socially isolating, and tedious hours during the early months of the pandemic resulted in a close-knit group of healthcare workers across the different COVID-19 treatment centres. The resulting sense of camaraderie and teamwork was seen as a key factor for healthcare workers to continue to provide healthcare services.

"We watch each other's back and help each other with tasks…this is one of the biggest successes we have seen during the period." (HCW 04, Doctor, Male)

Inexperienced healthcare workers, especially those recruited from other wards, were most appreciative of the close collaboration with senior colleagues as this improved their confidence.

For peer review only

"When I joined the treatment centre from another unit, I found it difficult to adapt... we had a lot of patients, and it was not easy…but our senior colleagues taught and supported us …. this really help me, and I became less afraid..." (HCW 03, Nurse, Female)

The healthcare workers also viewed collaborative and technical skills gained during COVID-19 patient care as making them more equipped and better prepared for future work.

"It is challenging but also a learning process as I have to deal with people from different sectors and backgrounds, not just the ones normally involved in the healthcare system..." (HCW 12, Doctor, Male)

"I have gained a lot of experience because I did not know how to use oxygen concentrators... but I can..." (HCW 03, Nurse, Female)

2.3. Training

Healthcare workers reported training centred around infection prevention and control (IPC) including the use of PPE and case management was the primary form of preparation for COVID-19 patient care. Training sessions were organised before the country's first COVID-19 case was reported on 31st March 2020 and this reduced anxiety over possible personal safety and healthcare worker infections. These training(s) also built on experiences gained during the 2014 Ebola outbreak which strengthened HCWs' competencies in IPC.

For peer review only - http://bmjopen.bmj.com/site/about/guidelines.xhtml

"We did refreshers trainings on how to put on PPE, IPC and hand hygiene before the COVID outbreak came into the country….so when COVID came we were not surprised…" (HCW 12, Doctor, Male)

"I think the Ebola gave us a footing especially with IPC…so it was not new…I think this is less stressful compared to the Ebola Era." (HCW 07, CHO, Female).

2.4. Sense of duty

All participants reported reminding themselves of their Hippocratic oath and the nature of their duties as a significant way of dealing with stress. Empathy towards the COVID-19 patients was also a strong motivator to continue patient care during the stressful period, with healthcare workers feeling unable to stay away and stop working despite the challenges and psychosocial outcomes.

"When I feel weak and don't want to go work I remember my nurse's and military pledge so I need to fulfil my duty…I can't stay home because you feel that it's your mom or daddy that is in the hospital bed, you need to go help…" (HCW 03, Nurse, Female)

2.5. 2014 Ebola outbreak

All the healthcare workers expressed that experiences gained while working during the 2014 Ebola outbreak were invaluable as they were more confident, better prepared psychologically and had transferable skills and competencies which reduced their levels of fear for providing care during the COVID-19 pandemic. Most participants reported feeling more afraid and stressed during the Ebola outbreak as compared to the COVID-19 pandemic demonstrating significant resilience, despite the presence of emotional triggers as previously described.

For peer review only - http://bmjopen.bmj.com/site/about/guidelines.xhtml

"The emotions during the Ebola was scarier than the COVID because that was the first outbreak exposure, COVID is my second so I did not have much fear to join the response because I had previous experience…" (HCW 13, Doctor, Male)

"The experience I got during Ebola was greater than this because during Ebola people were dying almost every minute…you needed great courage to work…we wore diapers inside the PPE, because if you left the ward you would not want to come back in…" (HCW 05, Nurse, Male)

3. Support structure

Frontline healthcare workers expressed vulnerability and resilience were influenced by their support structures.

3.1. Peer support

Gestures such as sharing meals, discussions before and after shifts as well as sharing jokes were expressed as the most common form of support. Several nurses expressed that encouraging words from doctors in charge of the COVID-19 unit also served as a boost. A WhatsApp group was used by some of the frontline healthcare workers to express their feelings, seek guidance and foster bonding among peers.

"We have this forum that everybody can vent out through audio clips, the experience of the day so we support each other and give advice on how to cope with the stress…" (HCW 10, Nurse, Female)

For peer review only - http://bmjopen.bmj.com/site/about/guidelines.xhtml

"When I feel weak…I listen to gospel songs…when I do this I feel Ok within myself…God gives me the energy and strength to be able to cope with the work." (HCW 06, Nurse, Female)

3.2. Donations

Donations from organisations and the community to COVID-19 treatment centres came in as medications, equipment, and personal items for COVID-19 patients, with a few targeting the healthcare workers. These gestures were received as a welcoming boost improving both patient care and morale among the staff.

"The day we received donations and had enough medications and some of the equipment we needed in the treatment centre…it lifted the mood of the entire staff, people were willing to work, absenteeism reduced…that was a big game changer." (HCW 12, Doctor, Male)

3.3. Professional support

Although there were psychosocial support services for patients in the treatment centres, this service is not accessible to frontline healthcare workers involved in the treatment of admitted COVID-19 patients. This was met with frustration from being excluded from such services by some healthcare workers.

"We have gone through the Ebola, mudslide, flooding now COVID, I know Sierra Leonean we are resilient, but we should not take it for granted…we are traumatised and full of anxiety and nobody cares about our emotional stress…" (HCW 10, Nurse, Female)

3.4. Health system challenges

For peer review only - http://bmjopen.bmj.com/site/about/guidelines.xhtml

Some participants expressed a sense of missed opportunity to capitalise on the country's history of outbreaks to strengthen its health system.

"During Ebola we had support from all over the world but with COVID-19, everyone has been grounded in their respective countries…this is a way of telling us to strengthen our health sector…" (HCW 13, Doctor, Male)

Improving conditions of service, availability of essential medications and supplies, the recruitment of volunteer healthcare workers not on the government payroll and the provision of health insurance for healthcare workers were repeatedly expressed.

"I think if we have the psychosocial counselling structure backed up by hospitals having the basics…this would help healthcare workers during outbreaks…" (HCW,11, Doctor, Male)

Discussion

This qualitative study explored the psychosocial experiences of frontline healthcare workers delivering COVID-19 care in Sierra Leone. The findings presented in this study are a particularly valuable in-depth qualitative analysis of psychosocial distress and extreme resilience among HCWs in the COVID-19 response. To date, the experiences of frontline healthcare workers who have been involved in both the 2014 West Africa Ebola outbreak and the COVID-19 pandemic are largely absent within the debate on psychosocial distress during epidemics. We present our conceptual framework Figure 1, to show that frontline healthcare workers' psychosocial outcomes in Sierra Leone during the COVID-19 outbreak, are shaped

For peer review only

by their vulnerabilities and resilience, with these experiences inexplicably linked to the strengths and weaknesses of the health system.

{INSET FIGURE 1}

Similar to other COVID-19 studies, our study also shows that stress, anxiety, fatigue, isolation, and fear of infection affected frontline HCWs the most during the start of the pandemic [35 36]. This is not surprising given the novelty of the disease and the traumatic nature of infectious disease outbreaks [37] We would therefore recommend that psychosocial support services be implemented early to promote resilience and protect healthcare workers.

Furthermore, studies done in sub-Saharan Africa, Asia and Europe during the COVID-19 pandemic emphasised similar themes to those we identified that the lack of medications, medical equipment and oxygen supply introduced further constraints in the workplace, affecting healthcare workers' ability to carry out their duties, frequently resulting in psychological distress [21 38 39]. The shortage of PPE was highlighted in systematic reviews as a major predisposing factor for negative psychological impact on healthcare workers during the COVID-19 pandemic [7 40]. However, our findings contradict this trend as HCWs did not express significant concerns in terms of the availability of PPE and were proud that the healthcare systems did not collapse. Despite this, the importance of PPE to healthcare workers in managing their fears and vulnerabilities was apparent and working with healthcare workers to achieve appropriate use of PPE based on disease conditions would support in ensuring efficient use of scarce health system resources. We recommend that investments in health system strengthening efforts should be prioritised as this is crucial in outbreak response to ensure the effectiveness of healthcare workers, improve safety and reduce stress [34].

For peer review only - http://bmjopen.bmj.com/site/about/guidelines.xhtml

Although all participants expressed psychological experiences, there were variations in the perceived levels based on gender, cadre and work experience. This study confirms findings from Ghana and the United Kingdom which highlighted that nurses are more vulnerable given their contact time with patients and in China which states that inexperienced healthcare workers express more stress while trying to adapt to the new settings [41-43]. Further explanation for the gender-related differences in our study may be because most of the female participants are nurses and males are doctors. Reflecting on this finding and considering gendered occupational segregation in the health workforce in Sierra Leone, with female nurses accounting for 90% of all nursing personnel [44]; gender considerations must be prioritised when planning psychosocial support services for healthcare workers, together with regular training and simulation exercises in emergency response to allay fear and anxiety among inexperienced healthcare workers during future outbreaks [22 27 36].

Our study adds to the overall body of evidence emerging from other studies on healthcare workers during outbreaks that direct involvement with patients had a varying effect on healthcare workers' relationships often resulting in stigmatisation increasing their psychological distress [3 12 35]. This signifies the need for the early introduction of risk communication and community engagement programs during outbreaks to increase awareness and reduce stigma.

Resilience among healthcare workers during the COVID-19 pandemic is well documented in the literature [38 45]. In our study, however, signs of resilience were mainly because of the legacy of the 2014 Ebola outbreak, a finding which is likely transferable to a limited number of contexts. The experience gained from patient care during the Ebola outbreak was seen as

For peer review only - http://bmjopen.bmj.com/site/about/guidelines.xhtml

training in outbreak response and formed part of healthcare workers' coping strategy. Our finding further showed that healthcare workers expressed optimism that the foundations laid by the Ebola outbreak have been built upon by the COVID-19 pandemic. Hence, they possess the necessary skills and expertise to face future outbreaks. This growing theme of resilience is also shown in the findings of a recent study to assess the impact of the COVID-19 pandemic on hospital utilisation in Sierra Leone [34]. It is worth acknowledging that despite the different nature and trajectory of both outbreaks with varying responses, there are common issues that inevitably shape the psychosocial outcomes of healthcare workers.

Measures such as training and availability of infection prevention and control measures have been found to have a pivotal impact in reducing frontline HCWs' risk of infections and a positive effect on their psychosocial outlook [3 4]. Findings from our study resonate with this body of evidence as multiple trainings were conducted for healthcare workers and the management of COVID-19 patients was separated from general care. As a result, participants felt safer, less anxious, and more confident to provide patient care [29]. Moving forward, training a critical mass of healthcare workers that possess the prerequisite knowledge, skills and competencies to provide quality healthcare to infectious disease patients that are admitted to a designated treatment centre could prove invaluable.

In line with previous studies done to assess motivators that kept HCWs working during outbreaks, we showed that the pandemic presented an opportunity for inexperienced frontline healthcare to learn new skills and develop stronger teams. Professional ethics, empathy, religion and community donations helped healthcare workers in coping with emotional struggles and physical exhaustion and focus on performing their duty [37-39].

For peer review only - http://bmjopen.bmj.com/site/about/guidelines.xhtml

Broader literature on support structures used by healthcare workers during outbreaks showed that HCWs discussed challenges and stresses with colleagues at the end of the shift and on social media platforms [4]. Our findings are in concordance with this peer support theme. However, the unavailability of counselling and professional support services for HCWs in our study resonates with findings from studies done in South Africa and Ghana but contrasts with those done in Europe where formal psychosocial support services were provided for healthcare workers [21 36 42]. This is disconcerting even with signs of resilience and success. It signifies a missed opportunity and gap presenting further evidence for the need to instil measures such as counselling services, the establishment of help hotlines and friendly relaxation spaces [46] to acknowledge and protect healthcare workers from undue exposure and adverse psychosocial outcomes during crises.

Psychosocial support structures must also go together with activities that strengthen the healthcare facilities given that the work environment is a strong predisposing factor for psychosocial distress. Furthermore, improvement of the conditions of service for healthcare workers with the introduction of health insurance schemes, employment of volunteering healthcare workers, and formal recognition of service and appraisal systems for frontline healthcare workers with exemplary performance during outbreak response would boost morale as well as reinforce positive conduct.

Strengths and Limitations

To the best of our knowledge, this is the first study to provide qualitative evidence of the psychosocial impacts of COVID-19 on healthcare workers in Sierra Leone. We interviewed different cadres of healthcare professionals across five designated COVID-19 treatment centres

For peer review only

which provided a depth of experiences and perspectives on our findings. The lead researcher (SMT) is a Sierra Leonean medical doctor and public health professional with established rapport, and connections and understands the local situation ensuring the trustworthiness of our findings.

There were some limitations. Our study was conducted in 2020 during the first wave of the outbreak, healthcare workers' perceptions and experiences may have evolved over this long pandemic. However, this study captured the experiences of healthcare workers at the height of the pandemic. Furthermore, recently published studies have highlighted similar experiences of healthcare workers that are involved in COVID-19 patient care [30 47].

The interviews were conducted remotely, and this may have influenced the degree to which healthcare workers may have responded. However, prior established rapport and communications with participants on social media and through emails before the interview process helped to circumvent this potential challenge. This is in keeping with recommendations from other qualitative studies that used remote interviewing [48 49]. This also allowed us to conduct interviews at the participant's preferred time, minimised distraction and was appropriate given the strict COVID-19 restrictions in place.

Conclusion

Our study is the first to explore the psychosocial impacts of COVID-19 among frontline healthcare workers in Sierra Leone. These different cadres of frontline healthcare workers experienced a wide range of psychosocial outcomes including anxiety, stress, guilt, anger, empathy, stigmatisation, and a sense of duty. Strong signs of resilience were also expressed

For peer review only - http://bmjopen.bmj.com/site/about/guidelines.xhtml

due to the legacy of the 2014 Ebola outbreak. We, therefore, recommend that strategies such as formal psychosocial support for healthcare workers, improved healthcare supply chain and the training of a critical mass of healthcare workers on outbreak preparedness and response be developed and implemented urgently to promote resilience and strengthen the health system in preparation for future epidemics.

Declarations

Acknowledgements

The authors would like to thank those who gave their time to participate and contribute to the study.

Contributors

SMT conceived the initial study and IFK, ST and LD contributed to study design and ethical approval process. SMT was responsible for data collection and was assisted by IFK, DBA, FFK and SS during this stage, SMT conducted data analysis alongside ST and LD. SMT produced the original draft of the manuscript. All authors critically reviewed and edited and provided comments on the first and subsequent drafts. All authors approved the final manuscript for submission.

Funding

This work was supported by the UK Research and Innovation (UKRI). The GCRF Accountability for Informal Urban Equity Hub ("ARISE") is a UKRI Collective Fund award with award reference ES/S00811X/1.

Competing interests

None declared

For peer review only

Patient consent for publication

Not required

Ethics approval

The study was reviewed and approved by the Sierra Leone Ethics and Scientific Review Committee (24072020). Written informed consent was obtained from all participants.

Data sharing statement

All data relevant to the study are included in the article.

Figure Legend:

Figure 1. Conceptual Framework showing the interlinkages that shape the psychosocial impact of COVID-19 on frontline healthcare workers in Sierra Leone, 2020.

Reference

1. Bloom DE, Cadarette D. Infectious Disease Threats in the Twenty-First Century: Strengthening the Global Response. *Frontiers in Immunology* 2019;10 doi: 10.3389/fimmu.2019.00549
2. Baker RE, Mahmud AS, Miller IF, et al. Infectious disease in an era of global change. *Nature Reviews Microbiology* 2022;20(4):193-205. doi: 10.1038/s41579-021-00639-z
3. Kisely S, Warren N, McMahon L, et al. Occurrence, prevention, and management of the psychological effects of emerging virus outbreaks on healthcare workers: rapid review and meta-analysis. *BMJ* 2020;369:m1642. doi: 10.1136/bmj.m1642
4. Billings J, Ching BCF, Gkofa V, et al. Experiences of frontline healthcare workers and their views about support during COVID-19 and previous pandemics: a systematic review and qualitative meta-synthesis. *BMC Health Services Research* 2021;21(1):923. doi: 10.1186/s12913-021-06917-z
5. Adams JG, Walls RM. Supporting the Health Care Workforce During the COVID-19 Global Epidemic. *Jama* 2020 doi: 10.1001/jama.2020.3972 [published Online First: 2020/03/13]
6. Lai J, Ma S, Wang Y, et al. Factors Associated With Mental Health Outcomes Among Health Care Workers Exposed to Coronavirus Disease 2019. *JAMA Netw Open*

For peer review only - http://bmjopen.bmj.com/site/about/guidelines.xhtml

2020;3(3):e203976. doi: 10.1001/jamanetworkopen.2020.3976 [published Online First: 2020/03/24]

7. Bekele F, Hajure M. Magnitude and determinants of the psychological impact of COVID-19 among health care workers: A systematic review. *SAGE Open Medicine* 2021;9:20503121211012512. doi: 10.1177/20503121211012512

8. Nuzzo JB, Meyer D, Snyder M, et al. What makes health systems resilient against infectious disease outbreaks and natural hazards? Results from a scoping review. *BMC Public Health* 2019;19 doi: 10.1186/s12889-019-7707-z

9. GOSL. National Ebola Recovery Strategy for Sierra Leone, 2015.

10. Evans DK, Goldstein M, Popova A. Health-care worker mortality and the legacy of the Ebola epidemic. *Lancet Glob Health* 2015;3(8):e439-e40. doi: 10.1016/s2214-109x(15)00065-0 [published Online First: 2015/07/15]

11. Shultz JM, Baingana F, Neria Y. The 2014 Ebola outbreak and mental health: current status and recommended response. *Jama* 2015;313(6):567-8. doi: 10.1001/jama.2014.17934 [published Online First: 2014/12/23]

12. Raven J, Wurie H, Witter S. Health workers' experiences of coping with the Ebola epidemic in Sierra Leone's health system: a qualitative study. *BMC Health Services Research* 2018;18(1):1-9. doi: doi:10.1186/s12913-018-3072-3

13. James PB, Wardle J, Steel A, et al. Providing healthcare to Ebola survivors: A qualitative exploratory investigation of healthcare providers' views and experiences in Sierra Leone. *Glob Public Health* 2020;15(9):1380-95. doi: 10.1080/17441692.2020.1762105 [published Online First: 2020/05/08]

14. McMahon SA, Ho LS, Brown H, et al. Healthcare providers on the frontlines: a qualitative investigation of the social and emotional impact of delivering health services during Sierra Leone's Ebola epidemic. *Health Policy Plan* 2016;31(9):1232-9. doi: 10.1093/heapol/czw055 [published Online First: 2016/06/10]

15. Wurie H, Witter S, Raven J. 'Fighting a battle': Ebola, health workers and the health system in Sierra Leone. 2016

16. World Health Organization. WHO Coronavirus (COVID-19) Dashboard 2022 [Available from: https://covid19.who.int/ accessed 11 May 2022 2022.

17. World Health Organization. Only 1 in 4 African health workers fully vaccinated against COVID-19 2021 [Available from: https://www.afro.who.int/news/only-1-4-african-health-workers-fully-vaccinated-against-covid-19 accessed 12 May 2021.

18. World Health Organization. Update on COVID-19 Sierra Leone 2022 [Available from: https://who.maps.arcgis.com/apps/dashboards/e5d453431c2143639d2ea3ef585e20a6.

19. Squire JS, Dadzie D, Nyarko KM, et al. Risk Factors for COVID-19 infection among Hospital Healthcare Workers, Sierra Leone, 2020. *Journal of Interventional Epidemiology and Public Health* 2022;5(4) doi: doi:10.11604/JIEPH.supp.2022.5.1.1198

20. Dean L, Cooper J, Wurie H, et al. Psychological resilience, fragility and the health workforce: lessons on pandemic preparedness from Liberia and Sierra Leone. *BMJ global health* 2020;5(9):e002873. doi: 10.1136/bmjgh-2020-002873

21. Aughterson H, McKinlay AR, Fancourt D, et al. Psychosocial impact on frontline health and social care professionals in the UK during the COVID-19 pandemic: a qualitative interview study. *BMJ Open* 2021;11(2):e047353. doi: 10.1136/bmjopen-2020-047353

For peer review only

For peer review only - http://bmjopen.bmj.com/site/about/guidelines.xhtml

22. Daneshvar E, Otterbach S, Alameddine M, et al. Sources of anxiety among health care workers in Tehran during the COVID-19 pandemic. *Health Policy and Planning* 2022;37(3):310-21. doi: 10.1093/heapol/czab136
23. Sun P, Wang M, Song T, et al. The Psychological Impact of COVID-19 Pandemic on Health Care Workers: A Systematic Review and Meta-Analysis. *Frontiers in Psychology* 2021;12 doi: 10.3389/fpsyg.2021.626547
24. Tessema GA, Kinfu Y, Dachew BA, et al. The COVID-19 pandemic and healthcare systems in Africa: a scoping review of preparedness, impact and response. *BMJ Global Health* 2021;6(12):e007179. doi: 10.1136/bmjgh-2021-007179
25. Gale NK, Heath G, Cameron E, et al. Using the framework method for the analysis of qualitative data in multi-disciplinary health research. *BMC Med Res Methodol* 2013;13:117. doi: 10.1186/1471-2288-13-117
26. Booth A, Hannes K, Harden A, et al. COREQ (Consolidated Criteria for Reporting Qualitative Studies). Guidelines for Reporting Health Research: A User's Manual2014:214-26.
27. Kamara IF, Tengbe SM, Fofanah BD, et al. Infection Prevention and Control in Three Tertiary Healthcare Facilities in Freetown, Sierra Leone during the COVID-19 Pandemic: More Needs to Be Done! *Int J Environ Res Public Health* 2022;19(9) doi: 10.3390/ijerph19095275 [published Online First: 2022/05/15]
28. Dietz PM, Centers for Disease Control and Prevention Ebola Response Group A, Georgia, Jambai A, et al. Epidemiology and Risk Factors for Ebola Virus Disease in Sierra Leone—23 May 2014 to 31 January 2015. *Clinical Infectious Diseases* 2015;61(11):1648-54. doi: 10.1093/cid/civ568
29. Kamara IF, Kumar AMV, Maruta A, et al. Antibiotic Use in Suspected and Confirmed COVID-19 Patients Admitted to Health Facilities in Sierra Leone in 2020-2021: Practice Does Not Follow Policy. *Int J Environ Res Public Health* 2022;19(7) doi: 10.3390/ijerph19074005 [published Online First: 2022/04/13]
30. May T, Aughterson H, Fancourt D, et al. 'Stressed, uncomfortable, vulnerable, neglected': a qualitative study of the psychological and social impact of the COVID-19 pandemic on UK frontline keyworkers. *BMJ Open* 2021;11(11):e050945. doi: 10.1136/bmjopen-2021-050945
31. DiCicco-Bloom B, Crabtree BF. The qualitative research interview. *Medical Education* 2006;40(4):314-21. doi: 10.1111/j.1365-2929.2006.02418.x
32. DeJonckheere M, Vaughn LM. Semistructured interviewing in primary care research: a balance of relationship and rigour. 2019 doi: 10.1136/fmch-2018-000057
33. Thompson A. Why Most of Africa's Data is Used on WhatsApp 2018 [Available from: https://theculturetrip.com/africa/articles/why-most-of-africas-data-is-used-on-whatsapp/ accessed 12 April 2020.
34. Sevalie S, Youkee D, van Duinen AJ, et al. The impact of the COVID-19 pandemic on hospital utilisation in Sierra Leone. *BMJ Global Health* 2021;6(10):e005988. doi: 10.1136/bmjgh-2021-005988
35. Sirois FM, Owens J. Factors Associated With Psychological Distress in Health-Care Workers During an Infectious Disease Outbreak: A Rapid Systematic Review of the Evidence. *Frontiers in Psychiatry* 2021;11 doi: 10.3389/fpsyt.2020.589545
36. Dawood B, Tomita A, Ramlall S. 'Unheard,' 'uncared for' and 'unsupported': The mental health impact of Covid -19 on healthcare workers in KwaZulu-Natal Province, South Africa. *PLOS ONE* 2022;17(5):e0266008. doi: 10.1371/journal.pone.0266008

For peer review only - http://bmjopen.bmj.com/site/about/guidelines.xhtml

37. Kisely S, Warren N, McMahon L, et al. Occurrence, prevention, and management of the psychological effects of emerging virus outbreaks on healthcare workers: rapid review and meta-analysis. 2020 doi: 10.1136/bmj.m1642
38. Shah J, Monroe-Wise A, Talib Z, et al. Mental health disorders among healthcare workers during the COVID-19 pandemic: a cross-sectional survey from three major hospitals in Kenya. *BMJ Open* 2021;11(6):e050316. doi: 10.1136/bmjopen-2021-050316
39. De Kock JH, Latham HA, Leslie SJ, et al. A rapid review of the impact of COVID-19 on the mental health of healthcare workers: implications for supporting psychological well-being. *BMC Public Health* 2021;21(1):104. doi: 10.1186/s12889-020-10070-3
40. Pappa S, Ntella V, Giannakas T, et al. Prevalence of depression, anxiety, and insomnia among healthcare workers during the COVID-19 pandemic: A systematic review and meta-analysis. *Brain Behav Immun* 2020 doi: 10.1016/j.bbi.2020.05.026
41. Liu Q, Luo D, Haase JE, et al. The experiences of health-care providers during the COVID-19 crisis in China: a qualitative study. *The Lancet Global Health* 2020;8(6):e790-e98. doi: 10.1016/S2214-109X(20)30204-7
42. Ofori AA, Osarfo J, Agbeno EK, et al. Psychological impact of COVID-19 on health workers in Ghana: A multicentre, cross-sectional study. *SAGE Open Medicine* 2021;9:20503121211000919. doi: 10.1177/20503121211000919
43. Lamb D, Gnanapragasam S, Greenberg N, et al. Psychosocial impact of the COVID-19 pandemic on 4378 UK healthcare workers and ancillary staff: initial baseline data from a cohort study collected during the first wave of the pandemic. *Occupational and Environmental Medicine* 2021;78(11):801. doi: 10.1136/oemed-2020-107276
44. World Health Organization. Sex distribution of health workers 2022 [Available from: https://apps.who.int/gho/data/node.main.HWFGRP_BYSEX?lang=en accessed 5 June 2022.
45. Kirabira J, Ben Forry J, Ssebuufu R, et al. Psychological distress and associated factors among hospital workers in Uganda during the COVID-19 lockdown – A multicentre study. *Heliyon* 2022;8(1):e08807. doi: https://doi.org/10.1016/j.heliyon.2022.e08807
46. Tomlin J, Dalgleish-Warburton B, Lamph G. Psychosocial Support for Healthcare Workers During the COVID-19 Pandemic. *Front Psychol* 2020;11:1960. doi: 10.3389/fpsyg.2020.01960 [published Online First: 2020/08/28]
47. Okediran JO, Ilesanmi OS, Fetuga AA, et al. The experiences of healthcare workers during the COVID-19 crisis in Lagos, Nigeria: A qualitative study. *Germs* 2020;10(4):356-66. doi: 10.18683/germs.2020.1228
48. Deakin H, Wakefield K. Skype interviewing: reflections of two PhD researchers:. *http://dxdoiorg/101177/1468794113488126* 2013 doi: 10.1177_1468794113488126
49. Johnson DR, Scheitle CP, Ecklund EH. Beyond the In-Person Interview? How Interview Quality Varies Across In-person, Telephone, and Skype Interviews. *Social Science Computer Review* 2019;39(6):1142-58. doi: 10.1177/0894439319893612

For peer review only

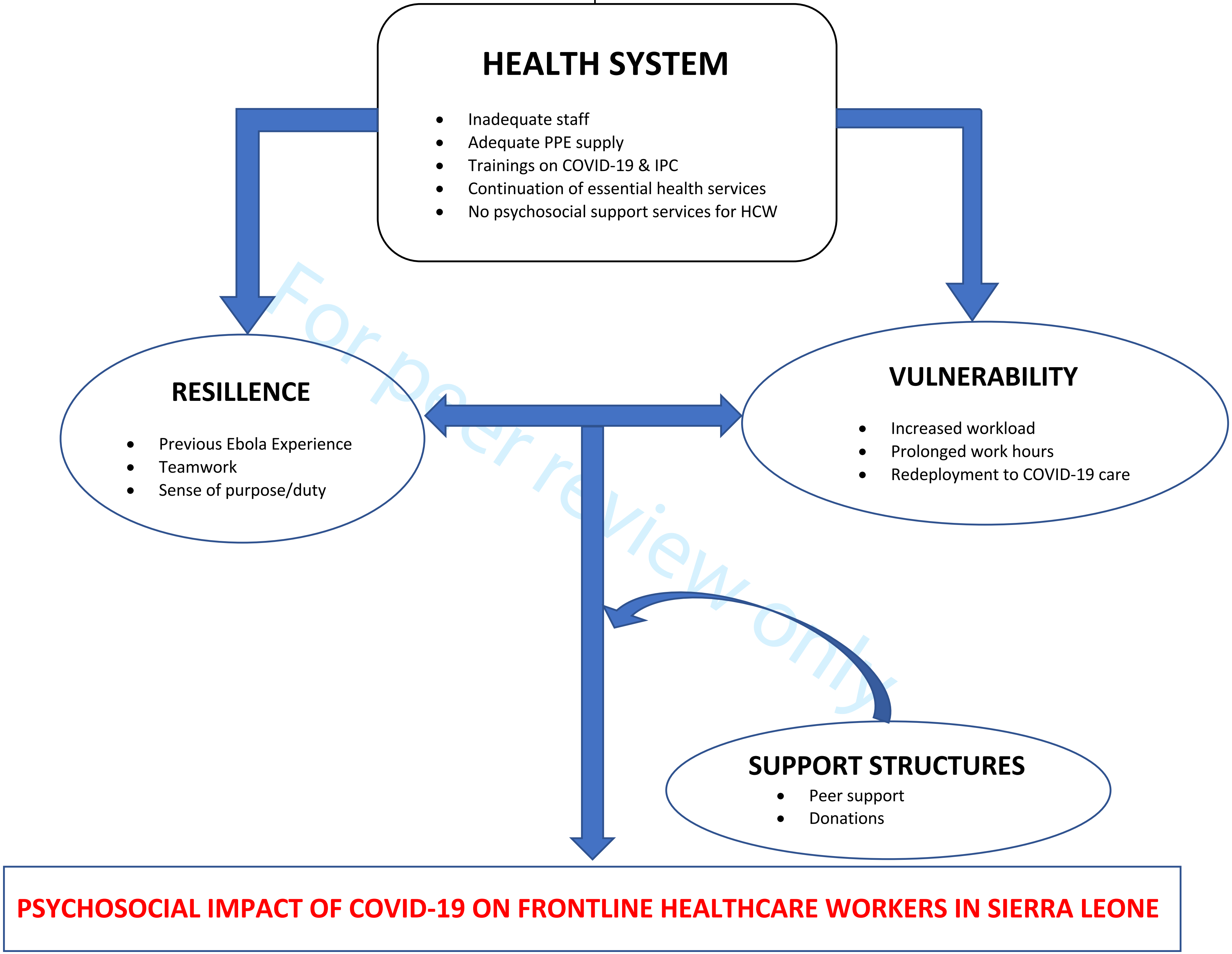

For peer review only - http://bmjopen.bmj.com/site/about/guidelines.xhtml

Topic Guide

A. Preliminary Note

This is the initial idea for the interview topic guide, and this will be extended and adjusted with growing insight into the research topic throughout the course of the research due to the iterative nature of qualitative research.

B. Introductions

Thank you very much for taking the time for this interview today. As outlined in the information sheet, our study aims to explore the psychosocial impacts of the COVID-19 pandemic and the Ebola outbreak on healthcare workers in Freetown Sierra Leone and develop recommendations for appropriate support. I am especially interested in your views and experiences as a frontline healthcare worker. Do you have any questions before we begin the interview?

C. Questions

1. *Life History*

- Would you tell be a bit about your professional background?
- What is your cadre as a healthcare worker?
- Are you employed by the government or by a non-governmental organization?
- What position do you hold in the organization?
- How long have you been working in the current COVID-19 response?
- What department did you working in prior to the COVID-19 response?

2. *Work Environment*

- Please tell me about your experiences in taking care of patients with COVID-19?
- How did you feel (emotions) on the first day?
- How are you feeling today?
- How do you feel when you get to work?
- What facilitates/ motivates you to give care to these patients?
- How prepared to you feel to provide care for COVID-19 patients?
- What challenges (emotional and resources) have you faced while providing care in the unit?
- How have you responded to the emotional challenges? (coping mechanisms)
- How has interactions with fellow healthcare workers been affected by working in the unit?
- What support (mental health/ psychological support) have you received?
- How do you feel about the support you have received? (assessible, efficient, preferences, shared decision making)
- How could the service provided be improved?

For peer review only

- Are there any other support (mental health /psychological support) would you have liked to have access to?
- Do you have any management roles/responsibilities? **(If No, skip to section 3)**
- Please tell me about your management experiences during COVID-19?
- How has COVID-19 pandemic affected your managerial duties?
- How has COVID-19 pandemic affected healthcare service provision within the facility?
- How prepared do you feel healthcare workers are to provide care for COVID-19 patients?
- What measures have you put in place to ensure healthcare workers are prepared to provide care for COVID-19 patients?
- What challenges (emotional and resources) do you feel frontline healthcare workers are facing while providing care in the unit?
- What challenges (emotional and resources) have frontline healthcare expressed?
- What do you feel is responsible for the different emotions' healthcare workers have expressed?
- How have you responded to the emotional challenges?
- What support (mental health/ psychological support) have you provided?
- How do you feel these support services have been received? (accessible/efficient/preference /shared decision making)
- How could the service provided be improved?
- Are there any other support (mental health /psychological support) would you like healthcare workers to have access to?
- Do you feel healthcare workers involved in the COVID-19 response should be recognized and appreciated? How?

3. *Family and Community?*

- Has working in this unit affected your interactions with family/ friends/ community?
- How has your family and community reacted to your work with COVID-19 patients?
- How have you responded to their reactions?
- Do you receive any support have from your family/ friends/community?

4. *Prior experience in outbreak?*

- Have you had any prior experience providing care/ in a managerial position during the Ebola outbreak?
- Can you share how you felt (experiences) while taking care of patients with Ebola
- How would you compare your emotional response providing care during Ebola to COVID-19 (similarities/differences)?
- Do you feel prior experience/exposure gained during the Ebola outbreak influence your reactions and feeling during the COVID-19 pandemic?

For peer review only

D. Concluding the interview

- Is there anything you would like to add to this discussion, or anything that we may have missed?
- *Give a recap of the discussion.*
- Do you have any questions you would like to ask me?
- Thank you very much for this interview and your time.

For peer review only

COREQ (COnsolidated criteria for REporting Qualitative research) Checklist

A checklist of items that should be included in reports of qualitative research. You must report the page number in your manuscript where you consider each of the items listed in this checklist. If you have not included this information, either revise your manuscript accordingly before submitting or note N/A.

Topic	Item No.	Guide Questions/Description	Reported on Page No.
**Domain 1: Research team and reflexivity**			
Personal characteristics			
Interviewer/facilitator	1	Which author/s conducted the interview or focus group?	
Credentials	2	What were the researcher's credentials? E.g. PhD, MD	
Occupation	3	What was their occupation at the time of the study?	
Gender	4	Was the researcher male or female?	
Experience and training	5	What experience or training did the researcher have?	
Relationship with participants			
Relationship established	6	Was a relationship established prior to study commencement?	
Participant knowledge of the interviewer	7	What did the participants know about the researcher? e.g. personal goals, reasons for doing the research	
Interviewer characteristics	8	What characteristics were reported about the inter viewer/facilitator? e.g. Bias, assumptions, reasons and interests in the research topic	
**Domain 2: Study design**			
Theoretical framework			
Methodological orientation and Theory	9	What methodological orientation was stated to underpin the study? e.g. grounded theory, discourse analysis, ethnography, phenomenology, content analysis	
Participant selection			
Sampling	10	How were participants selected? e.g. purposive, convenience, consecutive, snowball	
Method of approach	11	How were participants approached? e.g. face-to-face, telephone, mail, email	
Sample size	12	How many participants were in the study?	
Non-participation	13	How many people refused to participate or dropped out? Reasons?	
Setting			
Setting of data collection	14	Where was the data collected? e.g. home, clinic, workplace	
Presence of non-participants	15	Was anyone else present besides the participants and researchers?	
Description of sample	16	What are the important characteristics of the sample? e.g. demographic data, date	
Data collection			
Interview guide	17	Were questions, prompts, guides provided by the authors? Was it pilot tested?	
Repeat interviews	18	Were repeat inter views carried out? If yes, how many?	
Audio/visual recording	19	Did the research use audio or visual recording to collect the data?	
Field notes	20	Were field notes made during and/or after the inter view or focus group?	
Duration	21	What was the duration of the inter views or focus group?	
Data saturation	22	Was data saturation discussed?	
Transcripts returned	23	Were transcripts returned to participants for comment and/or	

For peer review only

Topic	Item No.	Guide Questions/Description	Reported on Page No.
		correction?	
**Domain 3: analysis and findings**			
Data analysis			
Number of data coders	24	How many data coders coded the data?	
Description of the coding tree	25	Did authors provide a description of the coding tree?	
Derivation of themes	26	Were themes identified in advance or derived from the data?	
Software	27	What software, if applicable, was used to manage the data?	
Participant checking	28	Did participants provide feedback on the findings?	
Reporting			
Quotations presented	29	Were participant quotations presented to illustrate the themes/findings? Was each quotation identified? e.g. participant number	
Data and findings consistent	30	Was there consistency between the data presented and the findings?	
Clarity of major themes	31	Were major themes clearly presented in the findings?	
Clarity of minor themes	32	Is there a description of diverse cases or discussion of minor themes?	

Developed from: Tong A, Sainsbury P, Craig J. Consolidated criteria for reporting qualitative research (COREQ): a 32-item checklist for interviews and focus groups. *International Journal for Quality in Health Care*. 2007. Volume 19, Number 6: pp. 349 – 357

Once you have completed this checklist, please save a copy and upload it as part of your submission. DO NOT include this checklist as part of the main manuscript document. It must be uploaded as a separate file.

For peer review only

BMJ Open

The Psychosocial Impact of COVID-19 Pandemic on Frontline Healthcare Workers in Sierra Leone: an explorative qualitative study

Journal:	*BMJ Open*
Manuscript ID	bmjopen-2022-068551.R2
Article Type:	Original research
Date Submitted by the Author:	19-Jun-2023
Complete List of Authors:	Tengbe, Sia Morenike; Sierra Leone Ministry of Health and Sanitation<br>Kamara, Ibrahim Franklyn; World Health Organisation Country Office for Sierra Leone, Emergency Preparedness and Response Cluster<br>Ali, Desta B; Sierra Leone Ministry of Health and Sanitation<br>Koroma, Fanny F; Sierra Leone Ministry of Health and Sanitation<br>Sevalie, Stephen; 34 Military Hospital, Wilberforce; Case Management Pillar, National COVID-19 Emergency Response Centre,<br>Dean, Laura; Liverpool School of Tropical Medicine, International Public Health<br>Theobald, Sally ; Liverpool School of Tropical Medicine, International Public Health
**Primary Subject Heading**:	Public health
Secondary Subject Heading:	Qualitative research, Mental health, Infectious diseases
Keywords:	COVID-19, Public health < INFECTIOUS DISEASES, MENTAL HEALTH

SCHOLARONE™
Manuscripts

BMJ

I, the Submitting Author has the right to grant and does grant on behalf of all authors of the Work (as defined in the below author licence), an exclusive licence and/or a non-exclusive licence for contributions from authors who are: i) UK Crown employees; ii) where BMJ has agreed a CC-BY licence shall apply, and/or iii) in accordance with the terms applicable for US Federal Government officers or employees acting as part of their official duties; on a worldwide, perpetual, irrevocable, royalty-free basis to BMJ Publishing Group Ltd ("BMJ") its licensees and where the relevant Journal is co-owned by BMJ to the co-owners of the Journal, to publish the Work in this journal and any other BMJ products and to exploit all rights, as set out in our licence.

The Submitting Author accepts and understands that any supply made under these terms is made by BMJ to the Submitting Author unless you are acting as an employee on behalf of your employer or a postgraduate student of an affiliated institution which is paying any applicable article publishing charge ("APC") for Open Access articles. Where the Submitting Author wishes to make the Work available on an Open Access basis (and intends to pay the relevant APC), the terms of reuse of such Open Access shall be governed by a Creative Commons licence – details of these licences and which Creative Commons licence will apply to this Work are set out in our licence referred to above.

Other than as permitted in any relevant BMJ Author's Self Archiving Policies, I confirm this Work has not been accepted for publication elsewhere, is not being considered for publication elsewhere and does not duplicate material already published. I confirm all authors consent to publication of this Work and authorise the granting of this licence.

For peer review only

The Psychosocial Impact of COVID-19 Pandemic on Frontline Healthcare Workers in Sierra Leone: an explorative qualitative study

Sia Morenike Tengbe [1], Ibrahim Franklyn Kamara [2], Desta B Ali [1], Fanny F Koroma [1], Stephen Sevalie [3, 4], Laura Dean [5], Sally Theobald [5]

1. Ministry of Health and Sanitation, Freetown, Sierra Leone
2. Emergency Preparedness and Response Cluster, World Health Organization Country Office, Freetown, Sierra Leone
3. 34 Military Hospital, Wilberforce, Freetown, Sierra Leone
4. Case Management Pillar, National COVID-19 Emergency Response Centre, Freetown, Sierra Leone
5. Department of International Public Health, Liverpool School of Tropical Medicine, Liverpool, UK

Correspondence to Sia Morenike Tengbe; siamoreniketengbe@outlook.com

Keywords: Psychosocial, Mental health, COVID-19, Healthcare workers, Sierra Leone.

For peer review only
For peer review only - http://bmjopen.bmj.com/site/about/guidelines.xhtml

Word count: 5143

Abstract

Introduction

The COVID-19 pandemic has wide-reaching health and non-health consequences, especially on mental health and psychosocial wellbeing. Healthcare workers involved in COVID-19 patient care are particularly vulnerable to psychosocial distress due to increased pressure on healthcare systems. We explored the psychosocial experiences of frontline healthcare workers during the COVID-19 pandemic in Sierra Leone.

Methods

This qualitative study used purposive sampling to recruit 13 healthcare workers from different cadres across five designated COVID-19 treatment centres in Freetown, Sierra Leone. In-depth interviews were conducted remotely in July and August 2020, transcribed verbatim and analysed using the framework approach.

Results

This study identified three overarching themes: vulnerability, resilience, and support structures. Participants expressed vulnerability relating to the challenging work environment and lack of medications as key stressors resulting in anxiety, stress, anger, isolation, and stigmatisation. Signs of resilience with experiences drawn from the 2014 Ebola outbreak, teamwork and a sense of duty were also seen. Peer support was the

For peer review only

main support structure with no professional psychosocial support services available to healthcare workers.

Conclusions

This is the first study to provide evidence of the psychosocial impacts of COVID-19 among frontline healthcare workers in Sierra Leone. Despite signs of resilience and coping mechanisms displayed, they also experienced adverse psychosocial outcomes. There is a need to focus on enhancing strategies such as psychosocial support for healthcare workers and those that overall strengthen the health system to protect healthcare workers, promote resilience, and guide recommendations for interventions during future outbreaks.

Article Summary

Strengths and Limitations of this study

- To the best of our knowledge, this is the first study to provide qualitative evidence of the psychosocial impacts of COVID-19 on healthcare workers in Sierra Leone.
- Interviewed different cadres of healthcare professionals across different COVID-19 treatment centres which provided a depth of experiences and perspectives on our findings.

For peer review only

- Our study was conducted in 2020 during the first wave of the outbreak, healthcare workers' perceptions and experiences may have evolved over this long pandemic.
- Interviews were conducted remotely, and this may have influenced the degree to which healthcare workers may have responded.

Introduction

Globally, we are experiencing an increasing trend of infectious disease outbreaks which have had a significant impact on livelihoods and public health and safety (1). Over the last two decades, the 2003 severe acute respiratory syndrome (SARS) outbreak, the 2009 Influenza (H1N1) pandemic, the 2012 Middle East respiratory syndrome coronavirus outbreak, the 2013–2016 Ebola virus disease epidemic in West Africa, the 2015 Zika virus disease and the ongoing COVID-19 pandemic have all had significant health and non-health impacts (2, 3).

Healthcare workers (HCWs) are a crucial resource in the management of outbreaks and are particularly vulnerable during these periods which brings additional challenges to their already stressful work environment including the increased risk of infection. The pre-existing work stress, coupled with additional outbreak-related stressors combined, may result in psychological distress amongst the health workforce especially pronounced in frontline HCWs directly involved in the diagnosis, treatment

For peer review only - http://bmjopen.bmj.com/site/about/guidelines.xhtml

and discharge of patients (4-7). It is therefore essential that policies and strategies that protect frontline healthcare workers and minimise their psychosocial distress are part of an effective public outbreak response.

In Sierra Leone, during the 2014 Ebola outbreak, healthcare workers were 30 times more likely to be infected than the public (8). This resulted in 295 healthcare worker infections and 221 deaths equating to a 6.85% loss of its total healthcare workforce (9, 10). Poor psychological outcomes including anxiety, post-traumatic stress disorder, anger, fear and community stigmatisation were reported amongst the few studies conducted during the period which focused on the outbreak's impact on healthcare workers (11, 12). Post-Ebola outbreak, negative psychological outcomes such as anxiety and anger still lingers among healthcare workers despite feeling more confident and knowledgeable in providing care for Ebola survivors (13).

Job-related stressors like increased workload, lack of personal protection equipment (PPE) and diagnostic capacity, prolonged shift hours, fear of possible infection and death of colleagues were shown to have a profound effect on healthcare workers' psychological wellbeing and coping mechanisms during the Ebola outbreak (12,14, 15).

Since the first cases of this novel coronavirus (severe acute respiratory syndrome coronavirus 2) emerged in Wuhan, China and the subsequent declaration of a

For peer review only

For peer review only - http://bmjopen.bmj.com/site/about/guidelines.xhtml

pandemic on 11th March 2020, over 550 million infections and 6.3 million deaths have been reported as of July 2022 (16). This is inclusive of more than 150,400 COVID-19 infections in healthcare workers in Africa and 269 in Sierra Leone (17,18). Furthermore, a recent study in a secondary hospital in Sierra Leone shows a high rate (29%) of secondary COVID-19 infections among healthcare workers (19).

Emerging evidence from the COVID-19 pandemic shows healthcare workers reporting high levels of psychological distress (20, 21). A systematic review and meta-analysis conducted during the first wave of the COVID-19 pandemic supports this evidence with higher levels of anxiety and depression reported among frontline healthcare workers (22). However, there is limited evidence from Sub-Saharan Africa focusing on the psychological impact of COVID-19 amongst healthcare workers in the region (23). In Sierra Leone, there are growing concerns that the pandemic may trigger anxiety and post-traumatic stress among healthcare workers similar to the devastating impact of the 2014 Ebola outbreak (24). Furthermore, studies conducted to assess COVID-19 preparedness in healthcare facilities in the country have shown HCWs perceive their facilities are ill-prepared to respond to the pandemic due to gaps in infrastructure and equipment including inadequate PPE (25, 26)

Given the gaps in the literature, this qualitative study aimed to explore the psychosocial experiences of frontline healthcare workers during the COVID-19 pandemic in Sierra Leone. The findings of this study could provide evidence for

For peer review only

recommendations to guide policy and practice and ensure support structures that build resilience among healthcare workers within the context of health systems in Sierra Leone are adopted during the COVID-19 pandemic and future outbreaks. Additionally, it will also add to the global, regional, and national body of evidence on the psychosocial impact of COVID-19 on healthcare workers.

Methodology

Study design

An explorative qualitative interview study underpinned by the naturalist research paradigm was conducted. The study drew on literature pertaining to resilience, trust and social support systems to inform our analytical process. Data were analysed using the framework approach, which supports the generation of themes and provided clear systematic steps while maintaining the transparency of the analytic process(27). The study is based on the COnsolidated criteria for REporting Qualitative research (COREQ) (28).

Study setting

This study was conducted in five out of the eight designated COVID-19 treatment centres in Freetown, the capital city of Sierra Leone and the epicentre of the COVID-19 outbreak (29). During the 2014 Ebola outbreak in Sierra Leone, Freetown was also one of the epicentres of the outbreak and recorded the highest prevalence (39%) of confirmed cases across the country (30).

For peer review only - http://bmjopen.bmj.com/site/about/guidelines.xhtml

For peer review only

In Sierra Leone, the management of COVID-19 patients was separated from regular health service provision to protect and maintain essential health services based on learnings from the 2014 Ebola outbreak. A total of 21 treatment centres were designated for COVID-19 across the country (31). These treatment centres were classified into three categories based on the severity of the disease. Isolation units (suspected cases), community care centres (asymptomatic and mild cases) and COVID-19 treatment centres (moderate, severe and critical cases, and all patients over 65 years with underlying medical conditions regardless of the severity of their symptoms) (31).

Sample and recruitment

We recruited thirteen frontline healthcare workers to complete in-depth interviews across five COVID-19 designated treatment centres in Freetown. Participants were interviewed in July and August 2020. Sampling was purposive to include a range of different cadres, employment statuses, gender, work experience and professional roles Table 1. All participants also had prior experience providing care during the 2014 Ebola outbreak. These participants were recruited via telephone calls, emails and social media networking platforms, using our pre-established communications and connections. Two of the researchers were members of the national COVID-19 response which made it easy to identify study participants and share study information with consented participants.

For peer review only - http://bmjopen.bmj.com/site/about/guidelines.xhtml

Table 1: Characteristics of frontline healthcare workers that participated in the study, July-August in Freetown, Sierra Leone, 2020

Participants	13
Cadres	Doctors (6)<br>Nurses (6)<br>Community Health Officer (1)
Gender	Male (7)<br>Female (6)
Employment status	Government (7)<br>Military (3)<br>Volunteers (3)
Work experience	5-10 years (11)<br>>10 years (2)
Location (work site)	COVID-19 treatment centres (CTC) (6)<br>Community care centres (CCC) (3)<br>Isolation units (2)<br>National COVID-19 Emergency Response Centre (NaCOVERC) (3)

Data collection

We conducted semi-structured, one-to-one interviews exploring the psychological and social impacts of the COVID-19 pandemic on participants. The interviews were conducted by SMT (lead researcher) remotely via Zoom or WhatsApp platforms using a pretested topic guide. The development of the topic guide was guided by existing behavioural change theories on health stress and coping mechanisms (32). This pretested topic guide has been provided as an online supplementary material. This

For peer review only

For peer review only - http://bmjopen.bmj.com/site/about/guidelines.xhtml

183 ensured we collected the information within a short time, minimising disruption to the
184 busy schedules of participants (33, 34). It was also appropriate given the COVID-19
185 social distancing restrictions which were in place (35, 36).

186 The interviews lasted for about 45-60 minutes and were done in Krio (local dialect) and
187 English (depending on participant preference) using a topic guide. All participants
188 were given a participant information sheet a week before the interviews and
189 encouraged to ask questions. Written informed consent was obtained prior to
190 interviews and a demographics form was completed by all participants. We audio-
191 recorded interviews with participants' consent, and recordings were transcribed by the
192 lead researcher (SMT) who has experience in qualitative research. Interviews
193 conducted in Krio were listened to and transcribed directly in English by the lead
194 researcher (ST) who is fluent in both languages. Where some words or concepts were
195 hard to understand or directly translate a third opinion from other researchers (IFK,
196 DBA and FFK) was sought as well as respondent validation. The audio recording of
197 interviews, interview notes and available transcripts enabled repeated revisiting of data
198 to remain immersed in the data and to participants' original accounts, helping to
199 enhance the validity of the results.

200 As this was a rapid study to provide evidence to support the ongoing COVID-19
201 response, interviews ceased when data saturation was reached as a commonality
202 between themes emerged and the lead author identified no new themes emerging
203 within the interviews.

For peer review only

Patient and public involvement

The study participants or the public were not involved in the design and conduct of the study. However, preliminary findings and recommendations have been shared with the case management pillar of the National COVID-19 Emergency Response centre and the Ministry of Health and Sanitation. Recommendations are designed to support improving the development of policy and practice to ensure healthcare workers are provided with psychosocial support during the pandemic and future disease outbreaks. The findings will be shared with the wider public and on social media.

For peer review only

Data Analysis

Following anonymisation by the lead researcher (SMT), transcripts were uploaded to NVivo Version 12 to aid analysis. Using the framework analysis approach, three researchers (SMT, ST and LD) initially read transcripts independently and discussed any emerging codes of potential significance to the research objectives. A coding framework was developed both deductively from the topic guide and study objectives, and inductively from themes emerging from the data. This coding framework was applied to each transcript with charts developed for each theme. All study authors agreed on the final themes and included rich and verbatim descriptions of participants' experiences to support these findings.

Study Trustworthiness

For peer review only - http://bmjopen.bmj.com/site/about/guidelines.xhtml

Respondent validation was used during the interview process to clarify responses; feeding back the information provided to participants ensured their responses were accurately captured and understood. This feedback process was conducted in more detail with interviews conducted in the local dialect to avoid misinterpretation even though the lead researcher is fluent in both languages to improve the credibility and confirmability of findings. Triangulation of findings from the data collection was also checked against secondary data from the literature to further aid credibility and transferability.

The positionality of the lead researcher (SMT) as a Sierra Leonean medical doctor and public health professional and with two other researchers (IFK and SS) being part of the COVID-19 response, ensured the establishment of rapport and mutual trust to aid data collection and to understand the local context and healthcare system. The methodological approach and findings were shared with key stakeholders to support dependability and our results include thick verbatim descriptions and quotes from participants to support our findings.

Results

The result is structured into three (3) key interlinking themes- vulnerability, resilience and support systems, each with several sub-themes Table 2.

For peer review only - http://bmjopen.bmj.com/site/about/guidelines.xhtml

Table 2: Themes and sub-themes of the psychosocial impacts of COVID-19 on frontline healthcare workers in Freetown, Sierra Leone, 2020.

THEME	SUB-THEME
1. Vulnerability	1.1. Work environment<br>1.2. Lack of resources and medication<br>1.3. Stigmatisation<br>1.4. PTSD<br>1.5. Lack of recognition
2. Resilience	2.1. Essential healthcare service provision<br>2.2. Teamwork<br>2.3. Training<br>2.4. Sense of purpose/duty<br>2.5. 2014 Ebola outbreak
3. Support Systems	3.1. Peer support<br>3.2. Donations<br>3.3. Professional support<br>3.4. Challenges in the health system

1. Vulnerability

1.1. Work environment

Despite acknowledging that healthcare workers are used to working under stressful conditions, increased experiences of stress, anxiety and isolation were expressed during COVID-19 patient care. These experiences peaked during the first three months

For peer review only

of the coronavirus pandemic and seemed to be more vividly expressed by females and nurses than by males and doctors.

"Working under stressful conditions is normal in Sierra Leone…" (HCW 08, Doctor, Male)

"When the first cases came, we were not enough at all…that time it was hell, because we had 30 beds and 4 nurses cannot deal with 30 patients…" (HCW 01, Nurse, Female)

Healthcare workers feeling overwhelmed were predominantly associated with disruptions in the work environment with increased workload, inadequate human resources, and a rapid influx of COVID-19 patients especially the critically ill.

The fear of possible infection due to patient care also fed into healthcare workers' vulnerability with concerns for personal safety and onward transmission to family members and friends.

"When a colleague became infected, it was really stressful…we did not know where she got the contact…we were quarantined, samples collected…we were scared…what if our samples turn out positive? what will we do? …we have put our lives and families at high risk…" (HCW 06, Nurse, Female).

For peer review only - http://bmjopen.bmj.com/site/about/guidelines.xhtml

1.2. Lack of resources and medications

Doctors and in-charge nurses with more managerial roles expressed anger, guilt, helplessness, and frustration related to the medical commodities such as medications, oxygen cylinders and personal protective equipment at COVID-19 treatment centres.

"The first death in the centre was diabetic and insulin was unavailable in the unit… it is challenging when you do not have what you need at the right time…. knowing at the back of your mind that if something goes wrong, you are to blame so it was very stressful."(HCW 08, Doctor, Male).

"We faced constraints with oxygen supply and if you have three patients in need it is difficult to know how to share it…I was monitoring a patient when I saw the oxygen cylinder was almost empty so I called and was told there was no gas…I was still on the call when the patient died… I just stood there…I felt guilty that there was something that could have been done…so it affected me for a few days…it ate at my conscience…" (HCW 01, Nurse, Female)

1.3. Stigmatisation

For peer review only

Stigmatisation either from colleagues not involved in the response and from the community resulted in HCWs feeling isolated and depressed. These feelings were equally expressed across all cadres and gender.

Our colleagues were scared for us, knowing that being at the frontline, we were at risk of becoming infected…in fact, they were also afraid of us, anytime we went to the regular hospital setting, they told us not to come close to them…" (HCW 02, Nurse, Female)

"When COVID-19 started, my family told me not to come home…they said I should not have joined the response…so I packed my things and went to stay in the hospital. I was in the hospital for over 1 month before I came home." (HCW 05, Nurse, Male)

1.4. Post-traumatic stress disorder symptoms

These findings also highlighted that the negative experiences from the 2014 Ebola outbreak such as fear and anxiety were triggered and led to some of the psychosocial outcomes frontline healthcare workers faced during the COVID-19 response. This was reflected in the inappropriate use of PPE despite limited supplies.

"The Ebola experience brought up some PTSD…when this corona started we observed that health workers were using too many PPE…because that was what they did during

For peer review only

the Ebola and the fear remembering that colleagues died from Ebola…" (HCW 11, Doctor, Male)

1.5. Lack of recognition

Frontline healthcare workers also reported feeling that their hard work was not adequately recognised, and appreciated by the government, especially the Ministry of Health and Sanitation. The lack of visitation by top government officials to COVID-19 treatment centres was mentioned by four healthcare workers as particularly discouraging.

"When the good comes, you hardly hear about the nurses… instead it is the big guns that are boasting…. what about the health workers that are in the fight…It's like nobody even cares…the minister has not even come around…those little things count." (HCW 10, Nurse, Female)

"A certificate of recognition should also be given to health workers in the frontline…this will help show that our efforts are valued and appreciated…" (HCW 13, Doctor, Male)

2. Resilience

Despite the challenges, pressures and vulnerabilities, frontline healthcare workers also demonstrated resilience, growth, pride, and job satisfaction during this period.

For peer review only

2.1. Essential healthcare services provision

The COVID-19 pandemic caused a significant shock to the health system and despite the challenges in the work environment and shortage of medications and commodities, medical doctors mainly viewed the COVID-19 response as a success as health services adapted, were maintained and did not collapse.

"In terms of successes, the health system has not collapsed…. We quickly move from 30-bed, decentralised, and have treatment facilities in almost every district…. I think we have 820 beds…. our surveillance is also good at pursuing contact…" (HCW 09, Doctor, Male)

"Sometimes we get shortage of PPE, but they fast track the process, so we have been able to maintain adequate PPE supplies, we have not reached the stage to recycle PPE…this has helped us to feel safe…" (HCW 04, Doctor, Male)

2.2. Teamwork

Working long, socially isolating, and tedious hours during the early months of the pandemic resulted in a close-knit group of healthcare workers across the different COVID-19 treatment centres. The resulting sense of camaraderie and teamwork was seen as a key factor for healthcare workers to continue to provide healthcare services.

"We watch each other's back and help each other with tasks…this is one of the biggest successes we have seen during the period." (HCW 04, Doctor, Male)

For peer review only - http://bmjopen.bmj.com/site/about/guidelines.xhtml

Inexperienced healthcare workers, especially those recruited from other wards, were most appreciative of the close collaboration with senior colleagues as this improved their confidence.

"When I joined the treatment centre from another unit, I found it difficult to adapt... we had a lot of patients, and it was not easy…but our senior colleagues taught and supported us …. this really help me, and I became less afraid..." (HCW 03, Nurse, Female)

The healthcare workers also viewed collaborative and technical skills gained during COVID-19 patient care as making them more equipped and better prepared for future work.

"It is challenging but also a learning process as I have to deal with people from different sectors and backgrounds, not just the ones normally involved in the healthcare system..." (HCW 12, Doctor, Male)

"I have gained a lot of experience because I did not know how to use oxygen concentrators... but I can..." (HCW 03, Nurse, Female)

2.3. Training

For peer review only - http://bmjopen.bmj.com/site/about/guidelines.xhtml

Healthcare workers reported training centred around infection prevention and control (IPC) including the use of PPE and case management was the primary form of preparation for COVID-19 patient care. Training sessions were organised before the country's first COVID-19 case was reported on 31st March 2020 and this reduced anxiety over possible personal safety and healthcare worker infections. These training(s) also built on experiences gained during the 2014 Ebola outbreak which strengthened HCWs' competencies in IPC.

"We did refreshers trainings on how to put on PPE, IPC and hand hygiene before the COVID outbreak came into the country....so when COVID came we were not surprised..." (HCW 12, Doctor, Male)

"I think the Ebola gave us a footing especially with IPC...so it was not new...I think this is less stressful compared to the Ebola Era." (HCW 07, CHO, Female).

2.4. Sense of duty

All participants reported reminding themselves of their Hippocratic oath and the nature of their duties as a significant way of dealing with stress. Empathy towards the COVID-19 patients was also a strong motivator to continue patient care during the stressful period, with healthcare workers feeling unable to stay away and stop working despite the challenges and psychosocial outcomes.

For peer review only - http://bmjopen.bmj.com/site/about/guidelines.xhtml

"When I feel weak and don't want to go work I remember my nurse's and military pledge so I need to fulfil my duty…I can't stay home because you feel that it's your mom or daddy that is in the hospital bed, you need to go help…" (HCW 03, Nurse, Female)

2.5. 2014 Ebola outbreak

All the healthcare workers expressed that experiences gained while working during the 2014 Ebola outbreak were invaluable as they were more confident, better prepared psychologically and had transferable skills and competencies which reduced their levels of fear for providing care during the COVID-19 pandemic. Most participants reported feeling more afraid and stressed during the Ebola outbreak as compared to the COVID-19 pandemic demonstrating significant resilience, despite the presence of emotional triggers as previously described.

"The emotions during the Ebola was scarier than the COVID because that was the first outbreak exposure, COVID is my second so I did not have much fear to join the response because I had previous experience…" (HCW 13, Doctor, Male)

"The experience I got during Ebola was greater than this because during Ebola people were dying almost every minute…you needed great courage to work…we wore diapers inside the PPE, because if you left the ward you would not want to come back in…" (HCW 05, Nurse, Male)

For peer review only - http://bmjopen.bmj.com/site/about/guidelines.xhtml

429 **3. Support structure**

430 Frontline healthcare workers expressed vulnerability and resilience were influenced by
431 their support structures.

432

433 3.1. Peer support

434 Gestures such as sharing meals, discussions before and after shifts as well as sharing
435 jokes were expressed as the most common form of support. Several nurses expressed
436 that encouraging words from doctors in charge of the COVID-19 unit also served as a
437 boost. A WhatsApp group was used by some of the frontline healthcare workers to
438 express their feelings, seek guidance and foster bonding among peers.

439

440 *"We have this forum that everybody can vent out through audio clips, the experience of*
441 *the day so we support each other and give advice on how to cope with the stress…" (HCW*
442 *10, Nurse, Female)*

443

444 *"When I feel weak…I listen to gospel songs…when I do this I feel Ok within myself…God*
445 *gives me the energy and strength to be able to cope with the work." (HCW 06, Nurse,*
446 *Female)*

447

448 3.2. Donations

449 Donations from organisations and the community to COVID-19 treatment centres
450 came in as medications, equipment, and personal items for COVID-19 patients, with a

For peer review only

few targeting the healthcare workers. These gestures were received as a welcoming boost improving both patient care and morale among the staff.

"The day we received donations and had enough medications and some of the equipment we needed in the treatment centre…it lifted the mood of the entire staff, people were willing to work, absenteeism reduced…that was a big game changer." (HCW 12, Doctor, Male)

3.3. Professional support

Although there were psychosocial support services for patients in the treatment centres, this service is not accessible to frontline healthcare workers involved in the treatment of admitted COVID-19 patients. This was met with frustration from being excluded from such services by some healthcare workers.

"We have gone through the Ebola, mudslide, flooding now COVID, I know Sierra Leonean we are resilient, but we should not take it for granted…we are traumatised and full of anxiety and nobody cares about our emotional stress…" (HCW 10, Nurse, Female)

3.4. Health system challenges

Some participants expressed a sense of missed opportunity to capitalise on the country's history of outbreaks to strengthen its health system.

For peer review only - http://bmjopen.bmj.com/site/about/guidelines.xhtml

"During Ebola we had support from all over the world but with COVID-19, everyone has been grounded in their respective countries…this is a way of telling us to strengthen our health sector…" (HCW 13, Doctor, Male)

Improving conditions of service, availability of essential medications and supplies, the recruitment of volunteer healthcare workers not on the government payroll and the provision of health insurance for healthcare workers were repeatedly expressed.

"I think if we have the psychosocial counselling structure backed up by hospitals having the basics…this would help healthcare workers during outbreaks…" (HCW,11, Doctor, Male)

Discussion

This qualitative study explored the psychosocial experiences of frontline healthcare workers delivering COVID-19 care in Sierra Leone. The findings presented in this study are a particularly valuable in-depth qualitative analysis of psychosocial distress and extreme resilience among HCWs in the COVID-19 response. To date, the experiences of frontline healthcare workers who have been involved in both the 2014 West Africa Ebola outbreak and the COVID-19 pandemic are largely absent within the debate on psychosocial distress during epidemics. We present our conceptual framework Figure

For peer review only - http://bmjopen.bmj.com/site/about/guidelines.xhtml

1, to show that frontline healthcare workers' psychosocial outcomes in Sierra Leone during the COVID-19 outbreak, are shaped by their vulnerabilities and resilience, with these experiences inexplicably linked to the strengths and weaknesses of the health system.

{INSET FIGURE 1}

Similar to other COVID-19 studies, our study also shows that stress, anxiety, fatigue, isolation, and fear of infection affected frontline HCWs the most during the start of the pandemic (37, 38). This is not surprising given the novelty of the disease and the traumatic nature of infectious disease outbreaks (39) We would therefore recommend that psychosocial support services be implemented early to promote resilience and protect healthcare workers.

Furthermore, studies done in sub-Saharan Africa, Asia and Europe during the COVID-19 pandemic emphasised similar themes to those we identified that the lack of medications, medical equipment and oxygen supply introduced further constraints in the workplace, affecting healthcare workers' ability to carry out their duties, frequently resulting in psychological distress (20, 40, 41). The shortage of PPE was highlighted in systematic reviews as a major predisposing factor for negative psychological impact on healthcare workers during the COVID-19 pandemic (7, 42). However, our findings contradict this trend as HCWs did not express significant concerns in terms of the

For peer review only - http://bmjopen.bmj.com/site/about/guidelines.xhtml

availability of PPE and were proud that the healthcare systems did not collapse. Despite this, the importance of PPE to healthcare workers in managing their fears and vulnerabilities was apparent and working with healthcare workers to achieve appropriate use of PPE based on disease conditions would support in ensuring efficient use of scarce health system resources. We recommend that investments in health system strengthening efforts should be prioritised as this is crucial in outbreak response to ensure the effectiveness of healthcare workers, improve safety and reduce stress (36).

Although all participants expressed psychological experiences, there were variations in the perceived levels based on gender, cadre and work experience. This study confirms findings from Ghana and the United Kingdom which highlighted that nurses are more vulnerable given their contact time with patients and in China which states that inexperienced healthcare workers express more stress while trying to adapt to the new settings (43-45). Further explanation for the gender-related differences in our study may be because most of the female participants are nurses and males are doctors. Reflecting on this finding and considering gendered occupational segregation in the health workforce in Sierra Leone, with female nurses accounting for 90% of all nursing personnel (46); gender considerations must be prioritised when planning psychosocial support services for healthcare workers, together with regular training and simulation exercises in emergency response to allay fear and anxiety among inexperienced healthcare workers during future outbreaks (21, 29, 38).

For peer review only

Our study adds to the overall body of evidence emerging from other studies on healthcare workers during outbreaks that direct involvement with patients had a varying effect on healthcare workers' relationships often resulting in stigmatisation increasing their psychological distress (3, 12, 37). This signifies the need for the early introduction of risk communication and community engagement programs during outbreaks to increase awareness and reduce stigma.

Resilience among healthcare workers during the COVID-19 pandemic is well documented in the literature (40, 47). In our study, however, signs of resilience were mainly because of the legacy of the 2014 Ebola outbreak, a finding which is likely transferable to a limited number of contexts. The experience gained from patient care during the Ebola outbreak was seen as training in outbreak response and formed part of healthcare workers' coping strategy. Our finding further showed that healthcare workers expressed optimism that the foundations laid by the Ebola outbreak have been built upon by the COVID-19 pandemic. Hence, they possess the necessary skills and expertise to face future outbreaks. This growing theme of resilience is also shown in the findings of a recent study to assess the impact of the COVID-19 pandemic on hospital utilisation in Sierra Leone (36). It is worth acknowledging that despite the different nature and trajectory of both outbreaks with varying responses, there are common issues that inevitably shape the psychosocial outcomes of healthcare workers.

For peer review only

Measures such as training and availability of infection prevention and control measures have been found to have a pivotal impact in reducing frontline HCWs' risk of infections and a positive effect on their psychosocial outlook (3, 4). Findings from our study resonate with this body of evidence as multiple trainings were conducted for healthcare workers and the management of COVID-19 patients was separated from general care. As a result, participants felt safer, less anxious, and more confident to provide patient care (31). Moving forward, training a critical mass of healthcare workers that possess the prerequisite knowledge, skills and competencies to provide quality healthcare to infectious disease patients that are admitted to a designated treatment centre could prove invaluable.

In line with previous studies done to assess motivators that kept HCWs working during outbreaks, we showed that the pandemic presented an opportunity for inexperienced frontline healthcare to learn new skills and develop stronger teams. Professional ethics, empathy, religion and community donations helped healthcare workers in coping with emotional struggles and physical exhaustion and focus on performing their duty (39-41).

Broader literature on support structures used by healthcare workers during outbreaks showed that HCWs discussed challenges and stresses with colleagues at the end of the shift and on social media platforms (4). Our findings are in concordance with this

For peer review only

peer support theme. However, the unavailability of counselling and professional support services for HCWs in our study resonates with findings from studies done in South Africa and Ghana but contrasts with those done in Europe where formal psychosocial support services were provided for healthcare workers (20, 38, 44). This is disconcerting even with signs of resilience and success. It signifies a missed opportunity and gap presenting further evidence for the need to instil measures such as counselling services, the establishment of help hotlines and friendly relaxation spaces (48) to acknowledge and protect healthcare workers from undue exposure and adverse psychosocial outcomes during crises.

Psychosocial support structures must also go together with activities that strengthen the healthcare facilities given that the work environment is a strong predisposing factor for psychosocial distress. Furthermore, improvement of the conditions of service for healthcare workers with the introduction of health insurance schemes, employment of volunteering healthcare workers, and formal recognition of service and appraisal systems for frontline healthcare workers with exemplary performance during outbreak response would boost morale as well as reinforce positive conduct.

Strengths and Limitations

To the best of our knowledge, this is the first study to provide qualitative evidence of the psychosocial impacts of COVID-19 on healthcare workers in Sierra Leone. We

For peer review only

For peer review only - http://bmjopen.bmj.com/site/about/guidelines.xhtml

interviewed different cadres of healthcare professionals across five designated COVID-19 treatment centres which provided a depth of experiences and perspectives on our findings.

There were some limitations. Our study was conducted in 2020 during the first wave of the outbreak, healthcare workers' perceptions and experiences may have evolved over this long pandemic. However, this study captured the experiences of healthcare workers at the height of the pandemic. Furthermore, recently published studies have highlighted similar experiences of healthcare workers that are involved in COVID-19 patient care (32, 49).

The interviews were conducted remotely, and this may have influenced the degree to which healthcare workers may have responded. However, prior established rapport and communications with participants on social media and through emails before the interview process helped to circumvent this potential challenge. This is in keeping with recommendations from other qualitative studies that used remote interviewing (50, 51). This also allowed us to conduct interviews at the participant's preferred time, minimised distraction and was appropriate given the strict COVID-19 restrictions in place.

Conclusion

Our study is the first to explore the psychosocial impacts of COVID-19 among frontline healthcare workers in Sierra Leone. These different cadres of frontline healthcare

For peer review only

For peer review only - http://bmjopen.bmj.com/site/about/guidelines.xhtml

workers experienced a wide range of psychosocial outcomes including anxiety, stress, guilt, anger, empathy, stigmatisation, and a sense of duty. Strong signs of resilience were also expressed due to the legacy of the 2014 Ebola outbreak. We, therefore, recommend that strategies such as formal psychosocial support for healthcare workers, improved healthcare supply chain and the training of a critical mass of healthcare workers on outbreak preparedness and response be developed and implemented urgently to promote resilience and strengthen the health system in preparation for future epidemics.

Declarations

Acknowledgements

The authors would like to thank those who gave their time to participate and contribute to the study.

Contributors

SMT conceived the initial study and IFK, ST and LD contributed to study design and ethical approval process. SMT was responsible for data collection and was assisted by IFK, DBA, FFK and SS during this stage, SMT conducted data analysis alongside ST and LD. SMT produced the original draft of the manuscript. All authors critically reviewed and edited and provided comments on the first and subsequent drafts. All authors approved the final manuscript for submission.

For peer review only - http://bmjopen.bmj.com/site/about/guidelines.xhtml

Funding

This work was supported by the UK Research and Innovation (UKRI). The GCRF Accountability for Informal Urban Equity Hub ("ARISE") is a UKRI Collective Fund award with award reference ES/S00811X/1. The write up was also supported by a Ken Newell Bursary award.

Competing interests

None declared

Patient consent for publication

Not required

Ethics approval

The study was reviewed and approved by the Sierra Leone Ethics and Scientific Review Committee (24072020) and Liverpool School of Tropical Medicine Masters Review Panel (20(02)a). Written informed consent was obtained from all participants.

Data sharing statement

All data relevant to the study are included in the article.

Figure Legend:

For peer review only - http://bmjopen.bmj.com/site/about/guidelines.xhtml

Figure 1. Conceptual Framework showing the interlinkages that shape the psychosocial impact of COVID-19 on frontline healthcare workers in Sierra Leone, 2020.

Reference

1. Bloom DE, Cadarette D. Infectious Disease Threats in the Twenty-First Century: Strengthening the Global Response. Frontiers in Immunology. 2019;10.

2. Baker RE, Mahmud AS, Miller IF, Rajeev M, Rasambainarivo F, Rice BL, et al. Infectious disease in an era of global change. Nature Reviews Microbiology. 2022;20(4):193-205.

3. Kisely S, Warren N, McMahon L, Dalais C, Henry I, Siskind D. Occurrence, prevention, and management of the psychological effects of emerging virus outbreaks on healthcare workers: rapid review and meta-analysis. BMJ. 2020;369:m1642.

4. Billings J, Ching BCF, Gkofa V, Greene T, Bloomfield M. Experiences of frontline healthcare workers and their views about support during COVID-19 and previous pandemics: a systematic review and qualitative meta-synthesis. BMC Health Services Research. 2021;21(1):923.

5. Adams JG, Walls RM. Supporting the Health Care Workforce During the COVID-19 Global Epidemic. Jama. 2020.

6. Lai J, Ma S, Wang Y, Cai Z, Hu J, Wei N, et al. Factors Associated With Mental Health Outcomes Among Health Care Workers Exposed to Coronavirus Disease 2019. JAMA Netw Open. 2020;3(3):e203976.

7. Bekele F, Hajure M. Magnitude and determinants of the psychological impact of COVID-19 among health care workers: A systematic review. SAGE Open Medicine. 2021;9:20503121211012512.

8. Nuzzo JB, Meyer D, Snyder M, Ravi SJ, Lapascu A, Souleles J, et al. What makes health systems resilient against infectious disease outbreaks and natural hazards? Results from a scoping review. BMC Public Health. 2019;19.

9. GOSL. National Ebola Recovery Strategy for Sierra Leone. 2015.

10. Evans DK, Goldstein M, Popova A. Health-care worker mortality and the legacy of the Ebola epidemic. Lancet Glob Health. 2015;3(8):e439-e40.

11. Shultz JM, Baingana F, Neria Y. The 2014 Ebola outbreak and mental health: current status and recommended response. Jama. 2015;313(6):567-8.

12. Raven J, Wurie H, Witter S. Health workers' experiences of coping with the Ebola epidemic in Sierra Leone's health system: a qualitative study. BMC Health Services Research. 2018;18(1):1-9.

13. James PB, Wardle J, Steel A, Adams J, Bah AJ, Sevalie S. Providing healthcare to Ebola survivors: A qualitative exploratory investigation of healthcare providers' views and experiences in Sierra Leone. Glob Public Health. 2020;15(9):1380-95.

14. McMahon SA, Ho LS, Brown H, Miller L, Ansumana R, Kennedy CE. Healthcare providers on the frontlines: a qualitative investigation of the social and emotional impact of

For peer review only

delivering health services during Sierra Leone's Ebola epidemic. Health Policy Plan. 2016;31(9):1232-9.
15. Wurie H, Witter S, Raven J. 'Fighting a battle': Ebola, health workers and the health system in Sierra Leone. 2016.
16. World Health Organization. WHO Coronavirus (COVID-19) Dashboard 2022 [Available from: https://covid19.who.int/.
17. World Health Organization. Only 1 in 4 African health workers fully vaccinated against COVID-19 2021 [Available from: https://www.afro.who.int/news/only-1-4-african-health-workers-fully-vaccinated-against-covid-19.
18. World Health Organization. Update on COVID-19 Sierra Leone 2022 [Available from: https://who.maps.arcgis.com/apps/dashboards/e5d453431c2143639d2ea3ef585e20a6.
19. Squire JS, Dadzie D, Nyarko KM, Danso-Appiah A, Kaburi BB, Noora CL, et al. Risk Factors for COVID-19 infection among Hospital Healthcare Workers, Sierra Leone, 2020. Journal of Interventional Epidemiology and Public Health. 2022;5(4).
20. Aughterson H, McKinlay AR, Fancourt D, Burton A. Psychosocial impact on frontline health and social care professionals in the UK during the COVID-19 pandemic: a qualitative interview study. BMJ Open. 2021;11(2):e047353.
21. Daneshvar E, Otterbach S, Alameddine M, Safikhani H, Sousa-Poza A. Sources of anxiety among health care workers in Tehran during the COVID-19 pandemic. Health Policy and Planning. 2022;37(3):310-21.
22. Sun P, Wang M, Song T, Wu Y, Luo J, Chen L, et al. The Psychological Impact of COVID-19 Pandemic on Health Care Workers: A Systematic Review and Meta-Analysis. Frontiers in Psychology. 2021;12.
23. Tessema GA, Kinfu Y, Dachew BA, Tesema AG, Assefa Y, Alene KA, et al. The COVID-19 pandemic and healthcare systems in Africa: a scoping review of preparedness, impact and response. BMJ Global Health. 2021;6(12):e007179.
24. Dean L, Cooper J, Wurie H, Kollie K, Raven J, Tolhurst R, et al. Psychological resilience, fragility and the health workforce: lessons on pandemic preparedness from Liberia and Sierra Leone. BMJ global health. 2020;5(9):e002873.
25. Kanu S, James PB, Bah AJ, Kabba JA, Kamara MS, Williams CEE, et al. Healthcare Workers' Knowledge, Attitude, Practice and Perceived Health Facility Preparedness Regarding COVID-19 in Sierra Leone. J Multidiscip Healthc. 2021;14:67-80.
26. Parmley LE, Hartsough K, Eleeza O, Bertin A, Sesay B, Njenga A, et al. COVID-19 preparedness at health facilities and community service points serving people living with HIV in Sierra Leone. PLOS ONE. 2021;16(4):e0250236.
27. Gale NK, Heath G, Cameron E, Rashid S, Redwood S. Using the framework method for the analysis of qualitative data in multi-disciplinary health research. BMC Med Res Methodol. 2013;13:117.
28. Booth A, Hannes K, Harden A, Noyes J, Harris J, Tong A. COREQ (Consolidated Criteria for Reporting Qualitative Studies). Guidelines for Reporting Health Research: A User's Manual2014. p. 214-26.
29. Kamara IF, Tengbe SM, Fofanah BD, Bunn JE, Njuguna CK, Kallon C, et al. Infection Prevention and Control in Three Tertiary Healthcare Facilities in Freetown, Sierra Leone during the COVID-19 Pandemic: More Needs to Be Done! Int J Environ Res Public Health. 2022;19(9).
30. Dietz PM, Centers for Disease Control and Prevention Ebola Response Group A, Georgia, Jambai A, Sierra Leone Ministry of Health and Sanitation Ebola Response Group F,

For peer review only

Paweska JT, National Institute for Communicable Diseases S-J, Gauteng, South Africa, et al. Epidemiology and Risk Factors for Ebola Virus Disease in Sierra Leone—23 May 2014 to 31 January 2015. Clinical Infectious Diseases. 2015;61(11):1648-54.

31. Kamara IF, Kumar AMV, Maruta A, Fofanah BD, Njuguna CK, Shongwe S, et al. Antibiotic Use in Suspected and Confirmed COVID-19 Patients Admitted to Health Facilities in Sierra Leone in 2020-2021: Practice Does Not Follow Policy. Int J Environ Res Public Health. 2022;19(7).

32. May T, Aughterson H, Fancourt D, Burton A. 'Stressed, uncomfortable, vulnerable, neglected': a qualitative study of the psychological and social impact of the COVID-19 pandemic on UK frontline keyworkers. BMJ Open. 2021;11(11):e050945.

33. DiCicco-Bloom B, Crabtree BF. The qualitative research interview. Medical Education. 2006;40(4):314-21.

34. DeJonckheere M, Vaughn LM. Semistructured interviewing in primary care research: a balance of relationship and rigour. 2019.

35. Thompson A. Why Most of Africa's Data is Used on WhatsApp 2018 [Available from: https://theculturetrip.com/africa/articles/why-most-of-africas-data-is-used-on-whatsapp/.

36. Sevalie S, Youkee D, van Duinen AJ, Bailey E, Bangura T, Mangipudi S, et al. The impact of the COVID-19 pandemic on hospital utilisation in Sierra Leone. BMJ Global Health. 2021;6(10):e005988.

37. Sirois FM, Owens J. Factors Associated With Psychological Distress in Health-Care Workers During an Infectious Disease Outbreak: A Rapid Systematic Review of the Evidence. Frontiers in Psychiatry. 2021;11.

38. Dawood B, Tomita A, Ramlall S. 'Unheard,' 'uncared for' and 'unsupported': The mental health impact of Covid -19 on healthcare workers in KwaZulu-Natal Province, South Africa. PLOS ONE. 2022;17(5):e0266008.

39. Kisely S, Warren N, McMahon L, Dalais C, Henry I, Siskind D. Occurrence, prevention, and management of the psychological effects of emerging virus outbreaks on healthcare workers: rapid review and meta-analysis. 2020.

40. Shah J, Monroe-Wise A, Talib Z, Nabiswa A, Said M, Abeid A, et al. Mental health disorders among healthcare workers during the COVID-19 pandemic: a cross-sectional survey from three major hospitals in Kenya. BMJ Open. 2021;11(6):e050316.

41. De Kock JH, Latham HA, Leslie SJ, Grindle M, Munoz S-A, Ellis L, et al. A rapid review of the impact of COVID-19 on the mental health of healthcare workers: implications for supporting psychological well-being. BMC Public Health. 2021;21(1):104.

42. Pappa S, Ntella V, Giannakas T, Giannakoulis VG, Papoutsi E, Katsaounou P. Prevalence of depression, anxiety, and insomnia among healthcare workers during the COVID-19 pandemic: A systematic review and meta-analysis. Brain Behav Immun. 2020.

43. Liu Q, Luo D, Haase JE, Guo Q, Wang XQ, Liu S, et al. The experiences of health-care providers during the COVID-19 crisis in China: a qualitative study. The Lancet Global Health. 2020;8(6):e790-e8.

44. Ofori AA, Osarfo J, Agbeno EK, Manu DO, Amoah E. Psychological impact of COVID-19 on health workers in Ghana: A multicentre, cross-sectional study. SAGE Open Medicine. 2021;9:20503121211000919.

45. Lamb D, Gnanapragasam S, Greenberg N, Bhundia R, Carr E, Hotopf M, et al. Psychosocial impact of the COVID-19 pandemic on 4378 UK healthcare workers and ancillary staff: initial baseline data from a cohort study collected during the first wave of the pandemic. Occupational and Environmental Medicine. 2021;78(11):801.

For peer review only

46. World Health Organization. Sex distribution of health workers 2022 [Available from: https://apps.who.int/gho/data/node.main.HWFGRP_BYSEX?lang=en.
47. Kirabira J, Ben Forry J, Ssebuufu R, Akimana B, Nakawuki M, Anyayo L, et al. Psychological distress and associated factors among hospital workers in Uganda during the COVID-19 lockdown – A multicentre study. Heliyon. 2022;8(1):e08807.
48. Tomlin J, Dalgleish-Warburton B, Lamph G. Psychosocial Support for Healthcare Workers During the COVID-19 Pandemic. Front Psychol. 2020;11:1960.
49. Okediran JO, Ilesanmi OS, Fetuga AA, Onoh I, Afolabi AA, Ogunbode O, et al. The experiences of healthcare workers during the COVID-19 crisis in Lagos, Nigeria: A qualitative study. Germs. 2020;10(4):356-66.
50. Deakin H, Wakefield K. Skype interviewing: reflections of two PhD researchers:. http://dxdoiorg/101177/1468794113488126. 2013.
51. Johnson DR, Scheitle CP, Ecklund EH. Beyond the In-Person Interview? How Interview Quality Varies Across In-person, Telephone, and Skype Interviews. Social Science Computer Review. 2019;39(6):1142-58.

For peer review only

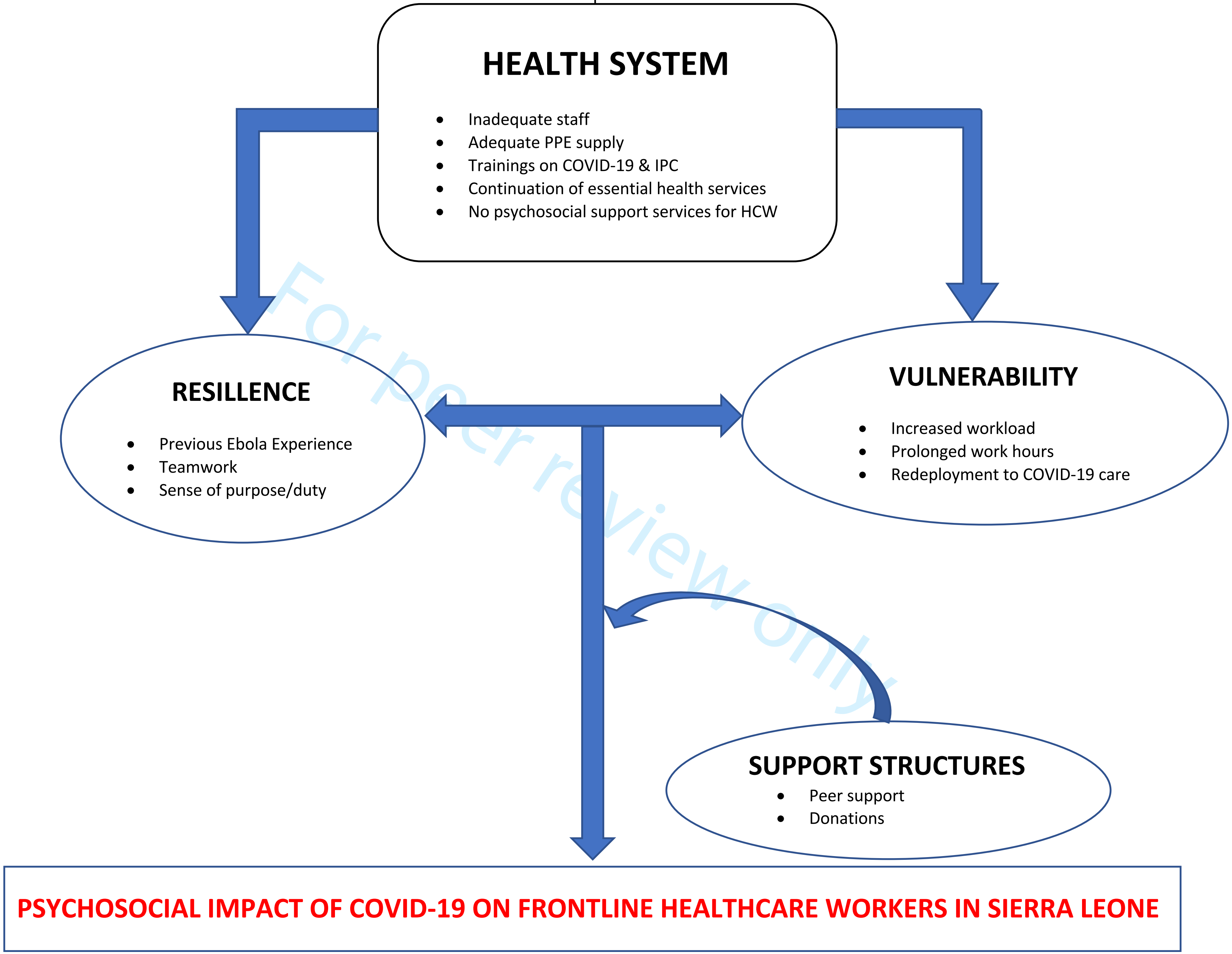

For peer review only

Topic Guide

A. Preliminary Note

This is the initial idea for the interview topic guide, and this will be extended and adjusted with growing insight into the research topic throughout the course of the research due to the iterative nature of qualitative research.

B. Introductions

Thank you very much for taking the time for this interview today. As outlined in the information sheet, our study aims to explore the psychosocial impacts of the COVID-19 pandemic and the Ebola outbreak on healthcare workers in Freetown Sierra Leone and develop recommendations for appropriate support. I am especially interested in your views and experiences as a frontline healthcare worker. Do you have any questions before we begin the interview?

C. Questions

1. *Life History*

- Would you tell be a bit about your professional background?
- What is your cadre as a healthcare worker?
- Are you employed by the government or by a non-governmental organization?
- What position do you hold in the organization?
- How long have you been working in the current COVID-19 response?
- What department did you working in prior to the COVID-19 response?

2. *Work Environment*

- Please tell me about your experiences in taking care of patients with COVID-19?
- How did you feel (emotions) on the first day?
- How are you feeling today?
- How do you feel when you get to work?
- What facilitates/ motivates you to give care to these patients?
- How prepared to you feel to provide care for COVID-19 patients?
- What challenges (emotional and resources) have you faced while providing care in the unit?
- How have you responded to the emotional challenges? (coping mechanisms)
- How has interactions with fellow healthcare workers been affected by working in the unit?
- What support (mental health/ psychological support) have you received?
- How do you feel about the support you have received? (assessible, efficient, preferences, shared decision making)
- How could the service provided be improved?

For peer review only

- Are there any other support (mental health /psychological support) would you have liked to have access to?
- Do you have any management roles/responsibilities? **(If No, skip to section 3)**
- Please tell me about your management experiences during COVID-19?
- How has COVID-19 pandemic affected your managerial duties?
- How has COVID-19 pandemic affected healthcare service provision within the facility?
- How prepared do you feel healthcare workers are to provide care for COVID-19 patients?
- What measures have you put in place to ensure healthcare workers are prepared to provide care for COVID-19 patients?
- What challenges (emotional and resources) do you feel frontline healthcare workers are facing while providing care in the unit?
- What challenges (emotional and resources) have frontline healthcare expressed?
- What do you feel is responsible for the different emotions' healthcare workers have expressed?
- How have you responded to the emotional challenges?
- What support (mental health/ psychological support) have you provided?
- How do you feel these support services have been received? (accessible/efficient/preference /shared decision making)
- How could the service provided be improved?
- Are there any other support (mental health /psychological support) would you like healthcare workers to have access to?
- Do you feel healthcare workers involved in the COVID-19 response should be recognized and appreciated? How?

3. *Family and Community?*

- Has working in this unit affected your interactions with family/ friends/ community?
- How has your family and community reacted to your work with COVID-19 patients?
- How have you responded to their reactions?
- Do you receive any support have from your family/ friends/community?

4. *Prior experience in outbreak?*

- Have you had any prior experience providing care/ in a managerial position during the Ebola outbreak?
- Can you share how you felt (experiences) while taking care of patients with Ebola
- How would you compare your emotional response providing care during Ebola to COVID-19 (similarities/differences)?
- Do you feel prior experience/exposure gained during the Ebola outbreak influence your reactions and feeling during the COVID-19 pandemic?

For peer review only

D. Concluding the interview

- Is there anything you would like to add to this discussion, or anything that we may have missed?
- *Give a recap of the discussion.*
- Do you have any questions you would like to ask me?
- Thank you very much for this interview and your time.

For peer review only

COREQ (COnsolidated criteria for REporting Qualitative research) Checklist

A checklist of items that should be included in reports of qualitative research. You must report the page number in your manuscript where you consider each of the items listed in this checklist. If you have not included this information, either revise your manuscript accordingly before submitting or note N/A.

Topic	Item No.	Guide Questions/Description	Reported on Page No.
**Domain 1: Research team and reflexivity**			
Personal characteristics			
Interviewer/facilitator	1	Which author/s conducted the interview or focus group?	
Credentials	2	What were the researcher's credentials? E.g. PhD, MD	
Occupation	3	What was their occupation at the time of the study?	
Gender	4	Was the researcher male or female?	
Experience and training	5	What experience or training did the researcher have?	
Relationship with participants			
Relationship established	6	Was a relationship established prior to study commencement?	
Participant knowledge of the interviewer	7	What did the participants know about the researcher? e.g. personal goals, reasons for doing the research	
Interviewer characteristics	8	What characteristics were reported about the inter viewer/facilitator? e.g. Bias, assumptions, reasons and interests in the research topic	
**Domain 2: Study design**			
Theoretical framework			
Methodological orientation and Theory	9	What methodological orientation was stated to underpin the study? e.g. grounded theory, discourse analysis, ethnography, phenomenology, content analysis	
Participant selection			
Sampling	10	How were participants selected? e.g. purposive, convenience, consecutive, snowball	
Method of approach	11	How were participants approached? e.g. face-to-face, telephone, mail, email	
Sample size	12	How many participants were in the study?	
Non-participation	13	How many people refused to participate or dropped out? Reasons?	
Setting			
Setting of data collection	14	Where was the data collected? e.g. home, clinic, workplace	
Presence of non-participants	15	Was anyone else present besides the participants and researchers?	
Description of sample	16	What are the important characteristics of the sample? e.g. demographic data, date	
Data collection			
Interview guide	17	Were questions, prompts, guides provided by the authors? Was it pilot tested?	
Repeat interviews	18	Were repeat inter views carried out? If yes, how many?	
Audio/visual recording	19	Did the research use audio or visual recording to collect the data?	
Field notes	20	Were field notes made during and/or after the inter view or focus group?	
Duration	21	What was the duration of the inter views or focus group?	
Data saturation	22	Was data saturation discussed?	
Transcripts returned	23	Were transcripts returned to participants for comment and/or	

For peer review only

Topic	Item No.	Guide Questions/Description	Reported on Page No.
		correction?	
**Domain 3: analysis and findings**			
Data analysis			
Number of data coders	24	How many data coders coded the data?	
Description of the coding tree	25	Did authors provide a description of the coding tree?	
Derivation of themes	26	Were themes identified in advance or derived from the data?	
Software	27	What software, if applicable, was used to manage the data?	
Participant checking	28	Did participants provide feedback on the findings?	
Reporting			
Quotations presented	29	Were participant quotations presented to illustrate the themes/findings? Was each quotation identified? e.g. participant number	
Data and findings consistent	30	Was there consistency between the data presented and the findings?	
Clarity of major themes	31	Were major themes clearly presented in the findings?	
Clarity of minor themes	32	Is there a description of diverse cases or discussion of minor themes?	

Developed from: Tong A, Sainsbury P, Craig J. Consolidated criteria for reporting qualitative research (COREQ): a 32-item checklist for interviews and focus groups. *International Journal for Quality in Health Care*. 2007. Volume 19, Number 6: pp. 349 – 357

Once you have completed this checklist, please save a copy and upload it as part of your submission. DO NOT include this checklist as part of the main manuscript document. It must be uploaded as a separate file.

For peer review only
